# Absence of Global Stress Regulation in *Escherichia coli* Promotes Pathoadaptation and Novel c-di-GMP-dependent Metabolic Capability

**DOI:** 10.1038/s41598-019-39580-w

**Published:** 2019-02-22

**Authors:** Nikola Zlatkov, Bernt Eric Uhlin

**Affiliations:** The Laboratory for Molecular Infection Medicine Sweden (MIMS), Department of Molecular Biology, Umeå University, Umea, Sweden

## Abstract

Pathoadaptive mutations linked to c-di-GMP signalling were investigated in neonatal meningitis-causing *Escherichia coli* (NMEC). The results indicated that NMEC strains deficient in RpoS (the global stress regulator) maintained remarkably low levels of c-di-GMP, a major bacterial sessility-motility switch. Deletion of *ycgG2*, shown here to encode a YcgG allozyme with c-di-GMP phosphodiesterase activity, and the restoration of RpoS led to a decrease in S-fimbriae, robustly produced in artificial urine, hinting that the urinary tract could serve as a habitat for NMEC. We showed that NMEC were skilled in aerobic citrate utilization in the presence of glucose, a property that normally does not exist in *E. coli*. Our data suggest that this metabolic novelty is a property of extraintestinal pathogenic *E. coli* since we reconstituted this ability in *E. coli* UTI89 (a cystitis isolate) *via* deactivation *rpoS*; additionally, a set of pyelonephritis *E. coli* isolates were shown here to aerobically use citrate in the presence of glucose. We found that the main reason for this metabolic capability is RpoS inactivation leading to the production of the citrate transporter CitT, exploited by NMEC for ferric citrate uptake dependent on YcgG2 (an allozyme with c-di-GMP phosphodiesterase activity).

## Introduction

Mutation and regulation are the two general, cynosural mechanisms of highly adaptive and evolutionary value to bacteria in times of trouble – value that can also result in different metabolic eventualities. Core metabolism is one of the basic determinants of bacterial species and is often underappreciated when bacterial pathogens are studied. *Escherichia coli*, for example, is a gammaproteobacterial species represented by commensal and pathogenic strains. Being facultative aerobes with a respiratory type and a fermentative type of metabolism, *E. coli* bacteria have strain-specific metabolic plasticity, which gives them the opportunity to utilize a large number of substrates as carbon and energy sources (e.g., D-glucose, D-mannitol, D-sorbitol, lactose, glycerol, tryptophan, lysine, and so on)^1^. The borders of the metabolic plasticity of *E. coli* are defined by the substrates that the bacteria cannot utilize, such as the conditional inability to use citrate^2,3^. This occurs firstly because *E. coli* cells express the CitT citrate transporter only under anaerobic conditions during which the tricarboxylic acid cycle is repressed, and second, citrate fermentation requires a reducing agent produced from a co-substrate (glucose or glycerol) to supply the citrate degradation pathway that eventually results in the production of succinate^2–4^. Some *E. coli* strains escape this scenario by acquiring plasmids (from *Salmonella typhi*) that carry the genes necessary for citrate utilization or *via* insertion of a promoter sequence that can trigger *citT* expression under aerobic conditions as a result of long-term evolution^5–8^.

Along with metabolic plasticity, the adaptive potential to abiotic stress of different *E. coli* groups defines their ability to thrive in restrictive niches. The group of extraintestinal pathogenic *E. coli* (ExPEC), to which neonatal meningitis-causing *E. coli* (NMEC) and uropathogenic *E. coli* (UPEC) belong, exhibits fitness that allows them to escape the gut and cause diseases elsewhere in the host^9,10^. *E. coli* O18:K1:H7 is a serotype typical of the bacterial agent causing newborn meningitis and early onset sepsis, diseases characterized by high mortality and morbidity among infants with severe implications on brain development, even if treated. To cause infection, NMEC cells escape the gut, survive in the blood and then, *via* transcytosis, cross the blood-brain barrier and colonize the brain tissue, which results in inflammation leading to meningitis^9–13^. Many virulence factors of ExPEC, including NMEC, strains are encoded on large, mobile genetic elements called pathogenicity islands (PAIs)^14–16^. The *sfa* gene cluster, found in 30% of the isolates, is located on such a genomic island and codes for the production of S-fimbriae, which are important components contributing to ExPEC virulence^17^. S-fimbriae are long, extracellular, filamentous organelles with a specific adhesin on their tips *via* which the bacteria adhere to sialylated glucoconjugates enriched in the brain tissue^17–19^. Even though NMEC serotypes have been found predominantly among neonatal meningitis isolates, they are also associated with sepsis and urinary tract infections^20–23^.

The pathogenic lifestyle of NMEC is also shaped by pathoadaptive mutations, *i.e*., changes in some of the core genes that increase the fitness of bacteria during the process of pathogenesis. A commonly occurring type of pathoadaptive mutations result in gene inactivation. Premature interruption of translation from transcripts of the *rpoS* gene is such an example. RpoS, a sigma factor acting in the RNA polymerase complex at the initiation of transcription, is the global stress regulator that provides cross-resistance against various abiotic stresses (such as deviations from optimal temperature, pH, osmotic pressure, against oxidative stress, and so on), and due to RpoS competition with the main vegetative sigma factor, RpoD, this cross-resistance comes at the expense of metabolic plasticity^24–28^. There are two groups of NMEC strains, those with active (e.g., *E. coli* C5) and inactive (e.g., *E. coli* IHE3034 and RS218) RpoS^29^, and the potential evolutionary reason underlying RpoS inactivation and the impact of this metabolic adaptation is an interest of this study.

Another type of gene alteration gives rise to new allelic variants that may result in active gene products. One example of such an allelic variant is found in the *ycgG* (*pdeG*) gene locus^30^. Gene alignment of IHE3034 against MG1655 revealed that a truncation of the *ycgG* reading frame resulted in a new *ycgG* allele, which led to the removal of the part coding for the membrane binding domain^30^. The *ycgG* gene is common among *E. coli* strains, and it is present in typical commensals, as represented by *E. coli* MG1655^31,32^. It codes for a membrane-bound c-di-GMP EAL phosphodiesterase (PDE) that breaks down c-di-GMP, the rather ubiquitous bacterial second messenger whose function is to serve as a sessility-motility switch, *i.e*., high levels of c-di-GMP trigger sessility and biofilm formation, while low levels of the messenger trigger motility^31,33^. The role of c-di-GMP was studied in some RpoS^+^ ExPEC strains, mainly in the *E. coli* CFT073 UPEC strain and to a lesser extent in RpoS^−^ NMEC *E. coli* IHE3034^34–38^. Overall, in *E. coli* CFT073, c-di-GMP represses type 1 fimbriae expression in a 9*pst* mutant variant in addition to triggering sessility and motility, and the presence of YdiV (a degenerated EAL phosphodiesterase) represses the formation of P-fimbriae^34–36^.

The versatile lifestyle of ExPEC variants prompted us to investigate the role of c-di-GMP and RpoS in the pathoadaptation of NMEC and other ExPEC strains.

## Results

### NMEC strains deficient in RpoS maintain low c-di-GMP levels

*E. coli* O18:K1:H7 strain IHE3034 is a neuroinvasive pathovar isolated in Finland in 1976 (referred to as IHE3034F in this study). After storage of the IHE3034F strain for a few decades under laboratory conditions, it represents a new strain variant (referred to as IHE3034) that has been studied extensively as an NMEC model strain^39^. Its biology, which combines the lack of RpoS with in the presence of YcgG2 and SfaY, together with the common ExPEC virulence-associated S-fimbriae, is what has made IHE3034 our model organism, and the role of c-di-GMP signalling its RpoS^−^ background was investigated.

**Figure 1:**
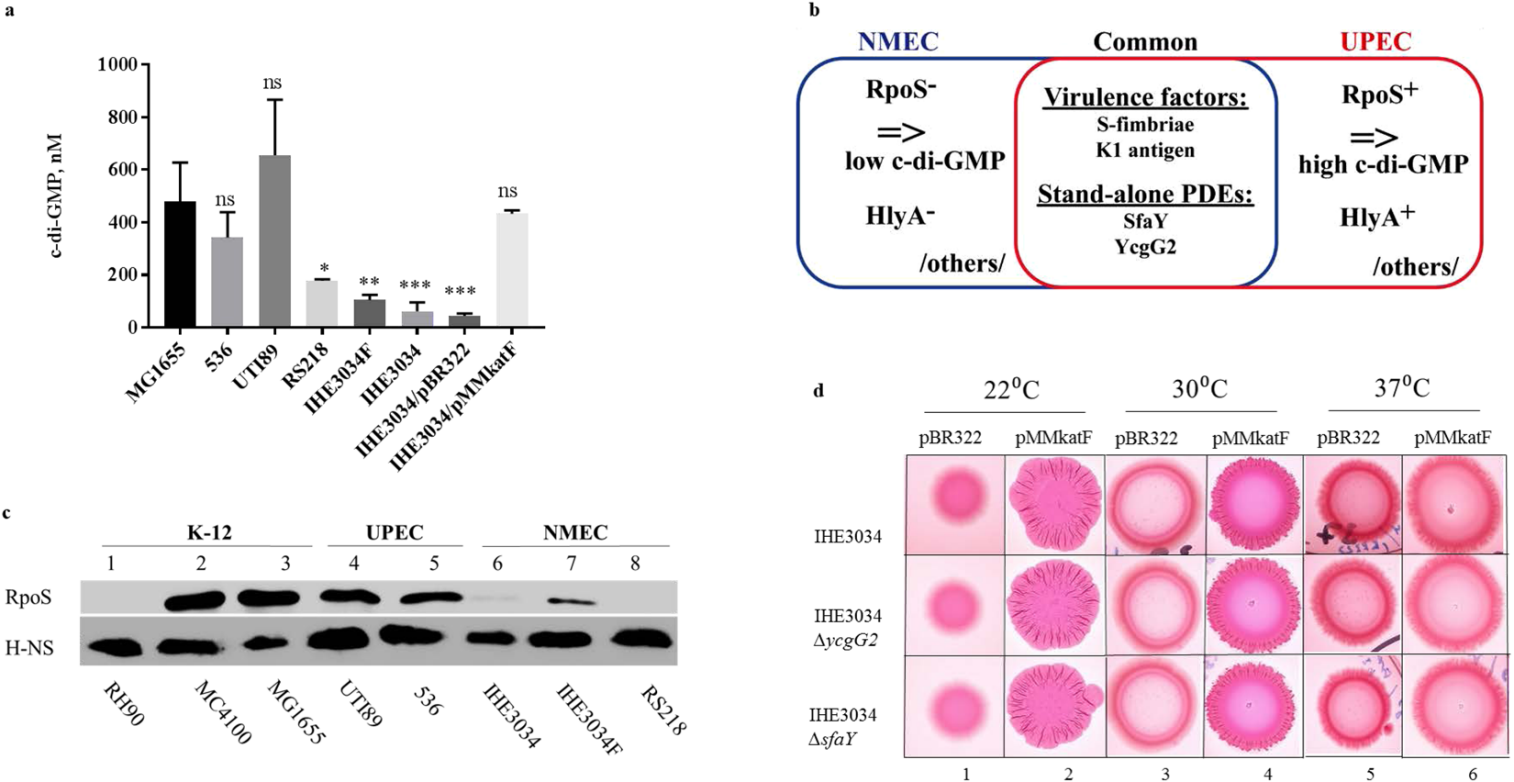
RpoS deficiency in NMEC. (**a**) Low c-di-GMP levels of NMEC strains (RS218, IHE3034F and IHE3034) compared to those of UPEC strains (536 and UTI89) due to RpoS deactivation. When RpoS activity was restored, the c-di-GMP levels of IHE3034 reached the levels of the K-12 MG1655 strain. (Data are represented as the mean +/− SD). (**b**) Schematic comparison between NMEC and UPEC. NMEC is schematically present in blue, while a UPEC cell that has the same K1 serotype (such as UTI89) of NMEC is shown in red. The common features are present in the intersection. (**c**) Immunoblotting against RpoS of different *E. coli* groups. Strain RH90 (MC4100*∆rpoS*) was used as a negative control. H-NS levels were used as a loading control. (**d**) Rugose colony formation by IHE3034 derivative strains with restored RpoS. Only the strains having pMMkatF showed rugose colony formation at 22 °C.

With the aim to investigate the role of c-di-GMP signalling in the lifestyle of NMEC, we measured the total c-di-GMP levels extracted from bacterial colonies produced by different ExPEC strains compared to levels produced by the commensal *E. coli* MG1655 (Fig. 1a). The NMEC strains (RS218, IHE3034F and IHE3034) showed unexpectedly low c-di-GMP levels, 5-10-fold lower than those of their close uropathogenic relatives UTI89 and 536, whose c-di-GMP levels were as high as those of the MG1655 K-12 commensal (Fig. 1a). NMEC and UPEC strains share many properties, presumably due to their on-going evolution and adaptation to extraintestinal niches (Fig. 1b). Nevertheless, one of the main differences between these two groups is the inactivation of the *rpoS* gene in NMEC bacteria (Fig. 1c), *i.e*., point mutations in the *rpoS* gene were observed in the IHE3034F strain that resulted in the expression of inactive RpoS (Fig. 1c; see lane 7 and Fig. S1), or in the genomes of other NMEC strains (such as RS218 and IHE3034), the *rpoS* gene was deactivated by the presence of a stop codon in the beginning of its ORF (Fig. 1c; see lanes 6 and 8 and Fig. S1). Since most experiments were performed with the IHE3034 strain, we undertook WGS of its original isolate, *i.e*., IHE3034F, and compared the two sequences. The WGS data showed that the genomes of IHE3034F and IHE3034 were very similar with slight changes depicted in Tables S1 and S2, suggesting that the IHE3034F strain did not change much during laboratory culture (Table S1 for indel mutations and Table S2 for SNPs). Neither of the differences are considered to influence the interpretation of the phenotypes studied in the present work. The sigma factor RpoS is the main global “player” that regulates c-di-GMP levels and turnover in *E. coli*. It activates a c-di-GMP “cascade” triggering the expression of curli fimbriae and cellulose by cells with high c-di-GMP levels, which produce rugose macrocolonies^31^. To test if restoration of RpoS activation in NMEC could bring back normal c-di-GMP levels and recover the rugose morphotype, we *trans*-complemented the IHE3034 isolate with an active *rpoS*^+^ allele. The RpoS^+^ strains exhibited the rugose morphotype (Fig. 1d; see row 2), and their c-di-GMP levels reached those of the control commensal strain MG1655 and of the tested UPEC strains (Fig. 1a), which also confirmed that RpoS production resulting from *trans-*complementation in NMEC reached the physiological levels normally produced in RpoS^+^ *E. coli*.

We considered that another reason for low c-di-GMP levels in some *E. coli* strains could be the expression of stand-alone EAL PDEs, which would cause a reduction in the c-di-GMP background. In the genome of IHE3034, two genes code for such enzymes – *sfaY* and *ycgG2*. We tested how mutations in these genes influenced the phenotypic effects of RpoS activity. RpoS restoration on the genetic background of these two mutants also resulted in the formation of rough colonies (Fig. 1d; see panel 2), which appeared identical to the colonies of the wild-type RpoS^+^ strain. Even though there are common features present in UPEC and NMEC, including virulence factors and EAL PDEs, our results suggested that inactive RpoS in NMEC is the main reason why this pathovar maintains much lower levels of c-di-GMP compared to those of its uropathogenic “kin” (Fig. 1b).

### A novel EAL phosphodiesterase from the *ycgG2* allele

**Figure 2:**
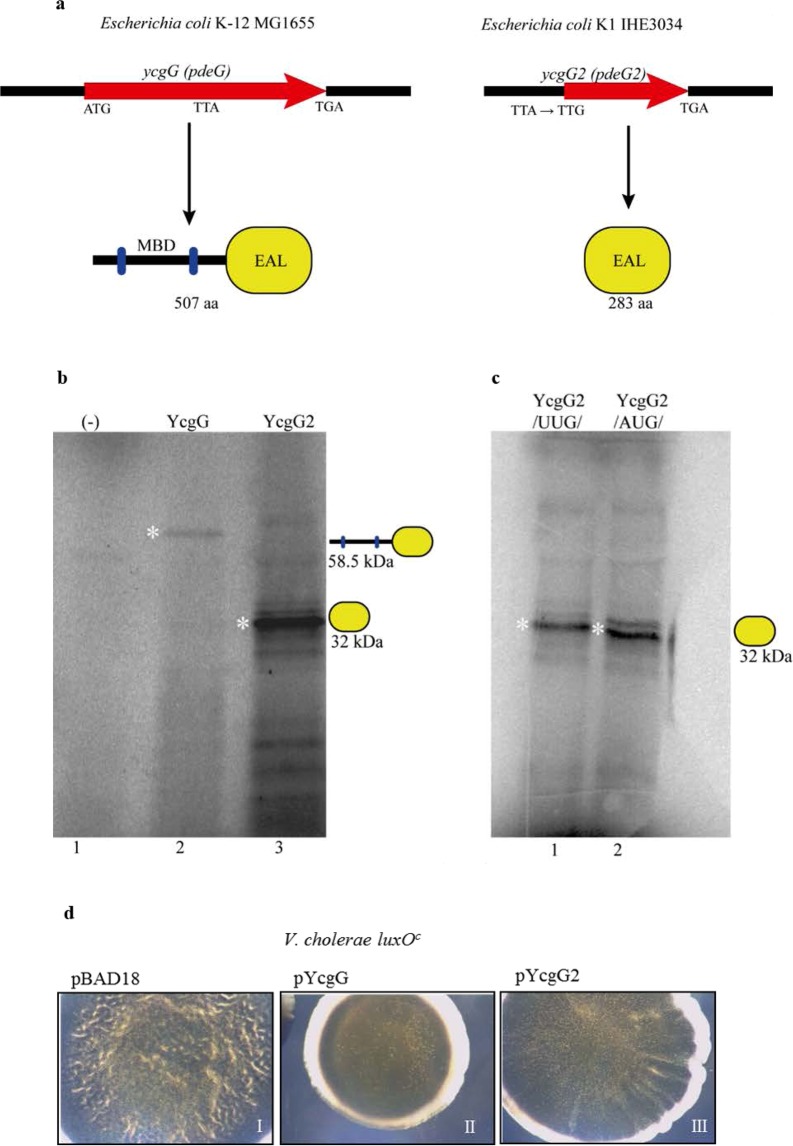
Properties of *ycgG2*. (**a**) Schematic comparison between *ycgG* and *ycgG2* and their products. Abbreviations: MBD – membrane-binding domain; EAL – a c-di-GMP PDE domain. (**b**) *In vitro* transcription/translation of *ycgG* (lane 2) and *ycgG2* (lane 3). Protein products indicated with a white star. The identity of YcgG2 was confirmed *via* ESI-MS analysis as described in the Methods. (**c**) *In vitro* transcription/translation of *ycgG2* from its native TTG start codon (lane 2) and from the ATG start codon (lane 3). Protein products indicated with a white star. (**d**) Ectopic expression of YcgG and Ycg2 in the *V. cholerae luxO*^c^ background.

Intriguingly, the *ycgG* gene is modified in the genome of NMEC (and of some other ExPEC strains belonging to the B2 group), giving rise to a new allelic variant^30^. In this new allelic version, here referred to as *ycgG2*, the portion of the ORF that encodes a putative membrane-binding domain was deleted, while the remaining first downstream codon was altered into a rare start codon (TTG), keeping the remaining part of the reading frame open and potentially coding only for the c-di-GMP PDE domain. These mutagenic events resulted in the possible production of YcgG2 as a second stand-alone PDE. Functionally, this variant would resemble SfaY, which earlier was shown to be a stand-alone PDE produced in NMEC from a gene located in the *sfa* gene cluster^38^. In addition, we observed that a point mutation in the first triplet of the leftover ORF turned it into a rare start codon and thus kept the remaining reading frame open (Fig. 2a). Alignment of their predicted protein sequences showed that the two sequences shared 98.2% similarity. The expected size of a predicted protein product from the *ycgG2* sequence was shown to be a 32-kDa 283-amino-acid protein (Fig. S2a and e). Previous attempts to confirm that the *ycgG* and *ycgG2* alleles encode the predicted proteins were unsuccessful^30^. It is also of interest to note the presence of one internal ATG codon at position 276, which has the potential to initiate translation in-frame, also preserving the EAL domain (Fig. S2a,c,d). To test if the *ycgG2* allele indeed is an ORF that can be translated into the predicted encoded product, we constructed expression operons with a T7 RNA polymerase promoter upstream of the *ycgG* and *ycgG2* genes and generated linear DNA fragments that were used as templates for *in vitro* transcription/translation in the PUREfrex 2.0 system composed of *E. coli* components (see Methods). Protein products were detected by [^35^S]-Met/Cys radioactive labelling. The results confirmed the coding potential of both the full-length *ycgG* and of the short allelic variant based on the presence of their 54-kDa and 32-kDa products, respectively (Fig. 2b; see lanes 2 and 3). To examine if the presence of TTG as a rare start codon in *ycgG2* has some adaptive advantage towards ribosome affinity, we exchanged the TTG codon with ATG and performed an *in vitro* assay comparing protein production to look for any alteration. The data indicated that the codon change did not cause any large difference in YcgG2 levels (Fig. 2c), and also, the presence of (p)ppGpp and c-di-GMP did not appear to affect ribosome affinity for the rare start codon (Fig. S2f and g), and thus YcgG2 levels remained the same.

Next, we aimed to determine if the *ycgG2* gene product, *i.e*., the YcgG2 protein, is an active PDE. We introduced *in-trans* the *ycgG2* and *ycgG* alleles on plasmid constructs in the *Vibrio cholerae luxO*^*c*^ strain, which exists in a mode allowing permanent biofilm formation due to the constant expression of LuxO, the main biofilm activator^40^; if c-di-GMP PDE activity is introduced in this strain, a rugose-smooth transition will be observed as a change in colony morphology. The results indicated the presence of phosphodiesterase activity exerted by both YcgG2 and YcgG (Fig. 2d). This verified YcgG2 as a new YcgG variant with reduced PDE activity (compared to that of YcgG), as shown by the intermediate smooth morphology of *V. cholerae luxO*^*c*^ colonies (Fig. 2d).

### S-fimbrial expression of IHE3034 bacteria is upregulated in artificial urine in a YcgG2 - dependent manner

**Figure 3:**
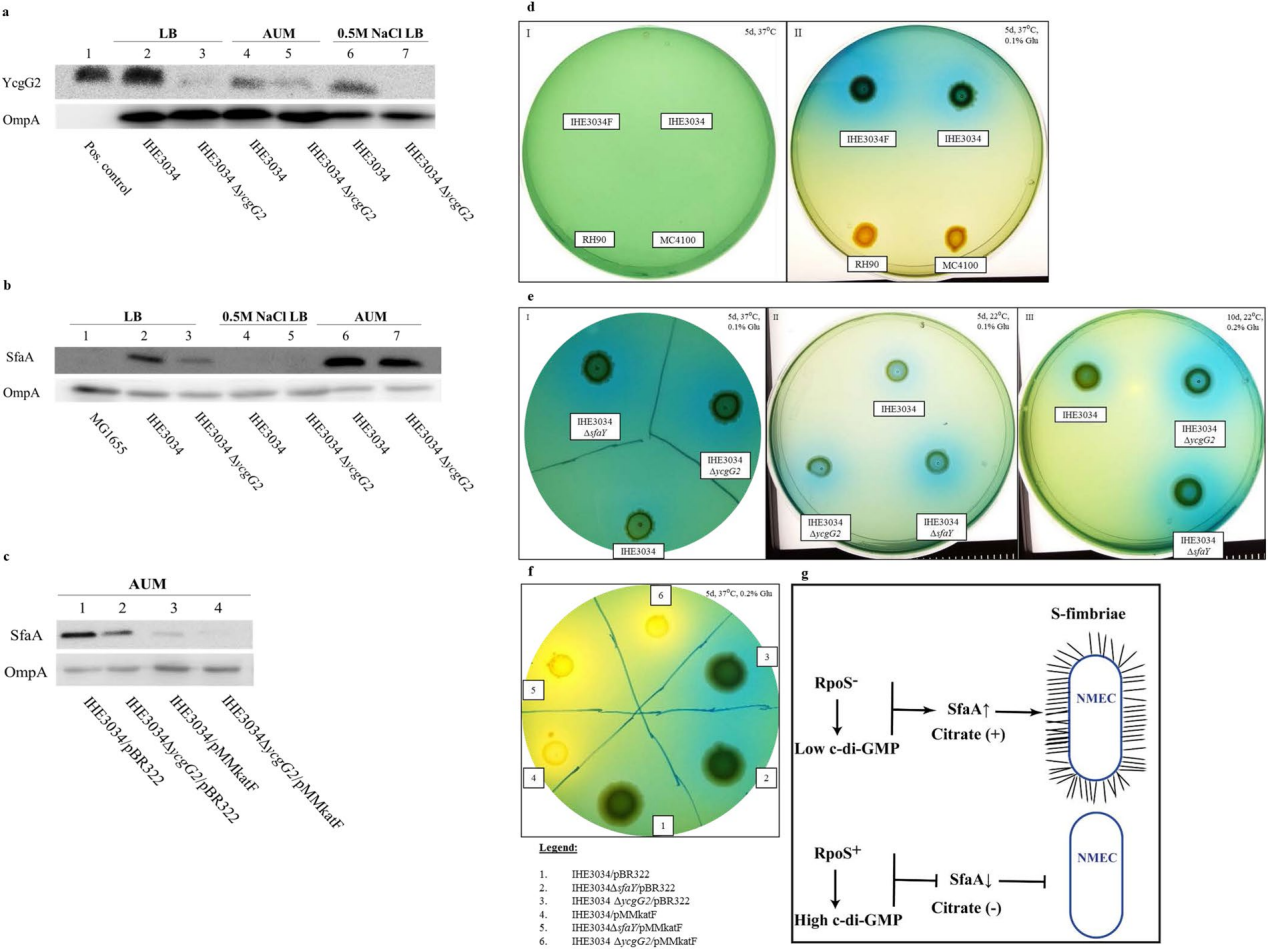
Effects of the absence of RpoS and YcgG2 on IHE3034 bacteria. For the citrate utilization assays, all conditions, i.e., days (d), temperature (°C) and percentage of glucose added (%), are stated in the upper corner of the subfigures. (**a**) YcgG2 levels of IHE3034 bacteria cultured in LB (sample 2), AUM (sample 4) and LB supplemented with 0.5 M NaCl (sample 6). Positive control – *in vitro*-produced YcgG2. ∆*ycgG2* bacterial extract, used as a negative control, showed very faint bands due to cross-reactivity of the antibody (samples 3 and 5). (**b**) SfaA levels of IHE3034 and ∆*ycgG2* bacteria cultured in LB, AUM and LB supplemented with 0.5 M NaCl. (**c**) SfaA levels of IHE3034 and ∆*ycgG2* bacteria with restored RpoS (strains 3 and 4, respectively) activity cultured in AUM compared to the levels of the empty-vector strains (strains 1 and 2). (**d**) Citrate utilization by IHE3034 cells. Box I: Lack of bacterial growth when citrate was provided as a single carbon and energy source. Box II: Citrate utilization by IHE3034 bacteria when glucose was provided as a co-substrate. Representatives of the rest of the strains utilizing only glucose, *i.e*., MC4100 and RH90 (MC4100*∆rpoS*), were used as negative controls. (**e**) Citrate utilization by IHE3034, ∆*ycgG2* and ∆*sfaY* bacteria on Simmon’s plates supplemented with 0.1% glucose and incubated at 37 °C (Box I) and at 22 °C (Box II), and with 0.2% glucose at 22 °C (Box III). (**f**) Citrate utilization by IHE3034, ∆*ycgG2* and ∆*sfaY* bacteria with restored RpoS activity compared to that of their empty-vector derivatives. (**g**) Schematic illustration of the effects of RpoS activity on S-fimbrial expression.

Most frequently, NMEC strains are transmitted vertically from mother to infant, and it is thought that the ability of the strains to reside and survive in the maternal urinary tract can allow such bacteria to be passed on later to the infant during birth. To test if the *ycgG2* allele might have some role in the adaptation to different growth conditions, as would occur during the different stages of infection, we incubated the IHE3034 wild-type and *∆ycgG2* mutant strains in artificial urine medium (AUM) or LB and in LB supplemented with 0.5 M NaCl. No differences in the growth rates of the two strains in these media were observed (Fig. S3a and b). The highest levels of YcgG2 were detected when the wild-type bacteria were grown in normal LB, but YcgG2 levels were somehow reduced when IHE3034 cells were cultured in the two hyperosmotic media (Fig. 3a; see lane 2 for LB, lane 4 for AUM and lane 6 for LB supplemented with 0.5 M NaCl).

Since NMEC evidently could grow well in AUM, we next tested if the bacteria could express the S-fimbriae that would contribute not only to survival in urine but also to colonization of the urinary tract. The levels of the SfaA major subunit were detected by western immunoblotting in samples from the wild-type and *∆ycgG2* mutant cells cultured in LB, AUM and LB with 0.5 M NaCl. The data revealed two main tendencies: when cultured in LB medium under optimal conditions, the IHE3034 cells produced SfaA in higher amounts compared to those of the *∆ycgG2* mutant cells (Fig. 3b; see lanes 2 and 3), while SfaA levels were high in both cases during incubation in AUM, and the lack of YcgG2 did not result in any detectable effect on the expression of the S-fimbrial major subunit (Fig. 3b; see lanes 6 and 7). On the other hand, the presence of higher concentrations of NaCl exerted an inhibitory effect on S-fimbrial production: for both strains, SfaA expression was below the limit of detection (Fig. 3b; see lanes 4 and 5). Since the production of SfaA was so high in AUM, we decided to test which component of the medium might be the signal sensed by bacteria. We incubated the IHE3034 bacteria in LB supplemented with different compounds used in AUM, and then we tested the expression of SfaA (Fig. S4). Neither of the components of the AUM managed to trigger S fimbriae biogenesis (Fig. S4, see lane 2 - positive control, lane 3–0.1% lactic acid, lane 4–0.04% iron (III) citrate, lane 5–0.17 M urea, lane 6–2.5 mM CaCl_2_, lane 7–0.002 M MnSO_4_, lane 8–0.002 M KCl and lane 9–0.013% ammonium sulfate).

The potential importance of lack of RpoS function for SfaA production was examined by using derivatives with restored *in-trans* RpoS activity that were incubated in AUM. The restored RpoS regulation had a clear negative effect on the expression of SfaA (Fig. 3c; see lane 3 and 4), which seemed further enhanced in the absence of YcgG2 (Fig. 3c; see lane 4). The data indicate that growth of NMEC in AUM was accompanied by higher levels of S-fimbriae production, which were somehow triggered by urine growth conditions, and we suggest that could be one of the reasons for RpoS deactivation in such isolates (Fig. 3g). We made an attempt to measure the levels of c-di-GMP of the *∆ycgG2* mutant bacteria incubated in LB overnight. Indeed, c-di-GMP levels were slightly higher than those of the wild-type strain, but the increase was statistically insignificant (17 nM c-di-GMP for the wild-type strain compared to 20 nM c-di-GMP for the mutant cells).

### RpoS inactivation results in metabolic reprogramming for citrate utilization in NMEC

RpoS inactivation and low c-di-GMP levels might open the door for some possible adaptive metabolic activity in the case of IHE3034 bacteria, mainly due to the lack of sigma factor competition between RpoD and RpoS^26,28^. We therefore explored if RpoS inactivation and expression of YcgG2 could lead to some new metabolic capacity that would increase the physiological fitness of IHE3034 bacteria in terms of utilization of alternative energy and carbon sources, for which *E. coli* has all the enzymes but shows differential expression. One of these potential substrates is citrate, which *E. coli* bacteria can utilize only under anaerobic conditions when a co-substrate such as glucose is provided^3^. We tested the aerobic citrate utilization of IHE3034 bacteria and the *∆ycgG2* mutant cells. For this purpose, we used Simmon’s agar^41^, in which citrate is in a complex with NH_4_^+^ in the presence of the pH indicator bromothymol blue (yellow when pH < 7, green when pH = 7 and blue when pH > 7). If citrate is utilized, only NH_4_^+^ will be left in the environment, as indicated by a blue halo around the bacterial colony. None of the bacterial strains grew aerobically on plain Simmon’s agar (Fig. 3d; see Box I), which suggested that either the CitT transporter was not expressed or that the required steps from the TCA cycle were not active. To provide an opportunity for the bacteria to grow on Simmon’s agar and to test which of the above reasons might be true, we embedded the medium with glucose on the surface of the agar and then incubated the strains again. This time, the blue halo and growth were considered to indicate a positive result. IHE3034 and IHE3034F bacteria gave positive results to citrate, while the K-12 strain MC4100 and its *rpoS* deletion mutant fed only on glucose and remained yellow and thus negative for citrate utilization (Fig. 3d; see Box II). Next, we checked if this metabolic feature could be dependent on c-di-GMP regulators. To increase the sensitivity of the bacteria to citrate and to check if this ability is temperature-dependent, we incubated the strains at 22 °C and 37 °C. Surprisingly, the *∆ycgG2* mutant colony gave a stronger positive result than the wild-type strain at both temperatures (Fig. 3e; see Box I, Box II and Box III and Fig. 3f; see strain 3). To determine if this behaviour was typical for downregulation of stand-alone PDE, we also tested if the *∆sfaY* mutant exhibited the same property under the same growth conditions. This deletion mutant showed the same mode of citrate utilization at both temperatures (Fig. 3e; see Box I, Box II and Box III, and Fig. 3f; see strain 2). To assess the role of the lost RpoS activity, we determined whether strains with restored RpoS, by *trans*-complementation, could utilize citrate in the same fashion as their parental strains (Fig. 3f). Only the YcgG2- and SfaY-deficient strains carrying the empty vector were able to utilize citrate (Fig. 3f; see strains 2 and 3), while RpoS^+^ strains were able to feed only on glucose (Fig. 3f; see strains 4, 5 and 6). To conclude, the absence of RpoS activity made IHE3034 bacteria capable of aerobic citrate utilization in the presence of glucose, an ability further regulated by YcgG2 and SfaY.

### Reprogramming for citrate utilization due to lack of RpoS is a specific property of extraintestinal pathogenic *E. coli*

**Figure 4:**
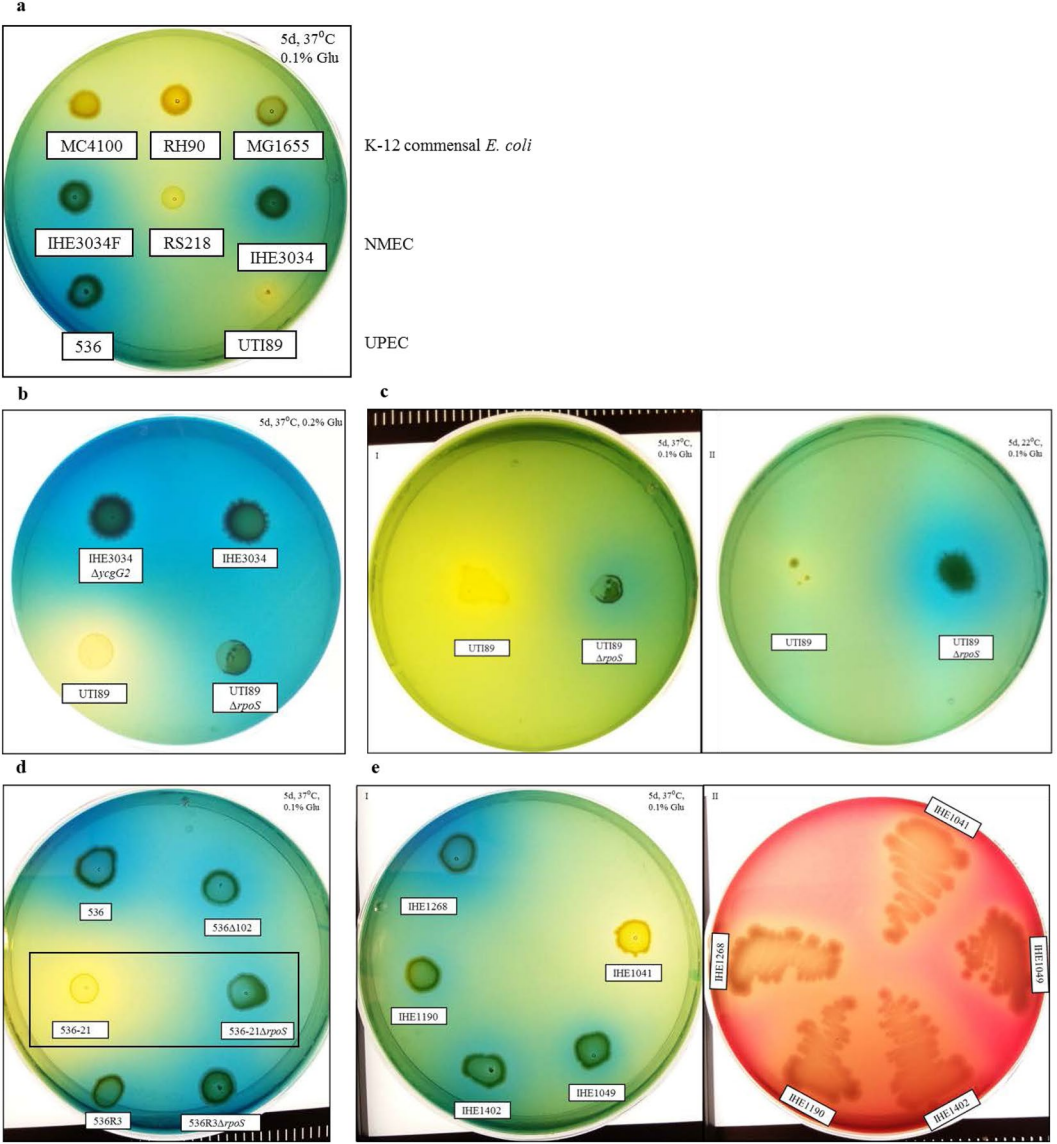
Aerobic citrate utilization by different ExPEC bacteria on Simmon’s plates when a co-substrate (glucose) is provided. All conditions, *i.e*., days (d), temperature (°C) and percentage of glucose added (%), are stated in the upper corner of the subfigures. (**a**) Citrate utilization by different *E. coli* groups, *i.e*., NMEC (IHE3034, IHE3034F and RS218), UPEC (UTI89 and 536), compared to that of the commensal MG1655. (**b**) Reconstitution of citrate uptake signalling events in the uropathogenic *E. coli* K1 strain UTI89. The wild-type UTI89 strain did not utilize citrate, while its RpoS-deficient variant (UTI89*∆rpoS*) showed positive results compared to those of IHE3034 and IHE3034 *∆ycgG2*. (**c**) UTI89 and UTI89*∆rpoS* bacteria tested for citrate utilization in the presence of 0.1% glucose at 37 °C (Box I) and at 22 °C (Box II). (**d**) The 536 pyelonephritis isolate, its less virulent derivatives, 536*∆*102 and 536R3, and its avirulent derivative, 536-21, were tested for citrate utilization. The 536-21 derivative tested negative, but its *∆rpoS* mutant was positive for citrate utilization (marked with black box), while the 536R3*∆rpoS* strain still tested positive for citrate, like its parental strain. (**e**) Different pyelonephritis clinical isolates tested for citrate utilization (Box I) and for haemolysis (Box II).

The finding that IHE3034 is able to use citrate when a co-substrate is provided prompted us to investigate how conserved this signalling mechanism is. We repeated again the screening on Simmon’s plates with glucose, and this time we included representatives of the commensal K-12 group (MG1655), of the UPEC group (the cystitis isolate strain UTI89 and the pyelonephritis isolate strain 536), and strain RS218 as another representative of the NMEC group. The results indicated that none of the commensal strains utilized citrate, while from the NMEC group, only IHE3034 and IHE3034F were able to use citrate, and strain RS218 remained negative for citrate utilization (Fig. 4a). An interesting observation was the fact that from the UPEC group, the cystitis isolate UTI89 was negative for citrate, while the pyelonephritis isolate 536 was positive (Fig. 4a).

Next, we tested if the signalling leading to aerobic citrate utilization could be restored in the strain UTI89, a cystitis isolate that is more closely related to IHE3034 than 536. UTI89 bacteria did not use citrate in Simmon’s medium supplemented with glucose, but when *rpoS* deletion was introduced, the UTI89*∆rpoS* bacteria, like IHE3034, started using citrate as a substrate in the presence of 0.2% (Fig. 4b) and 0.1% glucose (Fig. 4c, see Box I) at 37 °C. We also observed that the UTI89 bacteria were severely growth-retarded at 22 °C on Simmon’s medium supplemented with glucose, and interestingly, their growth ability was restored when the *rpoS* gene was deleted (Fig. 4b, see Box II).

Among the clinical isolates, the cystitis representative (UTI89) was negative for citrate utilization, while the pyelonephritis isolate, 536, gave a positive signal; thus, we next explored if citrate utilization could be linked to the virulence of this strain. To do so, we compared the citrate utilization of strain 536 to 536 derivatives that spontaneously lost their PAIs and/or their sites of integration (PAI I is the gene coding for tRNA^Sec^ and PAI II codes for tRNA^Leu^), which made them from less virulent, as for 536∆102 (PAI I^+^, PAI II^+^, *selC*^+^, *leuX*^−^), or avirulent, as for 536-21 (PAI I^−^, PAI II^−^, *selC*^−^, *leuX*^−^)^42–45^. As shown in Fig. 4d, all 536 derivatives were positive for citrate utilization, except 536-21, which, in addition to losing the two PAIs, also lost the tRNA^Sec^ and tRNA^Leu^ genes, reducing not only their virulence properties but their fitness in anaerobic growth^46^. To further test if citrate fermentation could be recovered in 536-21, we deleted the *rpoS* gene. When grown on Simmon’s supplemented with glucose, 536-21*∆rpoS* was able to feed on citrate and exhibited the same phenotypes as the IHE3034 and UTI89*∆rpoS* ExPEC strains (Fig. 4d, 536-21 and 536-21*∆rpoS* marked in black frame). Since the cystitis isolate UTI89 was negative for citrate utilization, while the pyelonephritis isolate was positive, we tested an array of pyelonephritis isolates to check if citrate utilization was their common feature. We tested strains IHE1041, IHE1049, IHE1190, IHE1268 and IHE1402, and 4 out of 5 were able to use citrate (Fig. 4e, see Box I), while all of them exhibited β-haemolysis (Fig. 4e, see Box II).

Overall, the results suggested that the signalling leading to citrate utilization is likely an ExPEC pathovar property rather than a specific NMEC feature since it could be observed in UPEC isolates, *i.e*., most of the tested pyelonephritis isolates (4 out of 5) were able to use citrate aerobically, and the cystitis isolate UTI89 became citrate-positive upon loss of RpoS, which was also the case with the RpoS-deficient NMEC strain IHE3034.

### CitT serves as a portal for citrate and iron (III) citrate

**Figure 5:**
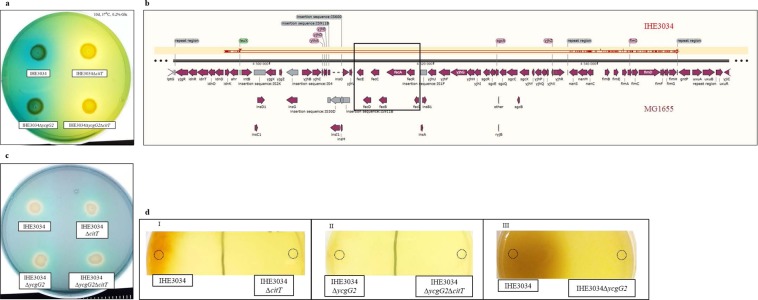
Analysis of IHE3034 bacteria for Fe(III) uptake. (**a**) Citrate utilization by IHE3034 and IHE3034∆*ycgG2* bacteria compared to that of their *∆citT* mutant derivatives. (**b**) Graphic representation of the local alignment of the sequence (shown in red) of the IHE3034 genome (coordinates 4927723–4959109) carrying the *fecIRfecABCDE* deletion against the genome of MG1655. Deletion of the operons in the IHE3034 genome is indicated with a black frame. (**c**) The general ability of IHE3034 and its ∆*citT*, ∆*ycgG2* and ∆*ycgG2*∆*citT* mutants to undergo Fe(III) acquisition, as tested on a CAS plate. The yellow halo around the bacteria indicated a positive reaction for siderophore production. (**d**) Fe(III) citrate utilization assay (sites of inoculation marked with rings). Box I: Fe(III) citrate utilization by IHE3034 compared to that of its ∆*citT* mutant. Box II: Fe(III) citrate utilization by IHE3034 ∆*ycgG2* compared to that of its ∆*citT* mutant. Box III: Reduced Fe(III) citrate utilization by wild-type IHE3034∆*ycgG2* compared to that of wild-type IHE3034.

We hypothesized that CitT might be expressed aerobically and that the phenotype indicating citrate uptake was accomplished through this transport system. To directly test if CitT was required, we introduced deletions in the *citT* genes of the IHE3034 and IHE3034*∆ycgG2* strains. Compared to their parental strains, the CitT-deficient strains were not able to utilize citrate (Fig. 5a), which suggested that endogenous *citT* gene expression was aerobically induced in the absence of RpoS and that CitT served as the only importer for citrate.

**Figure 6:**
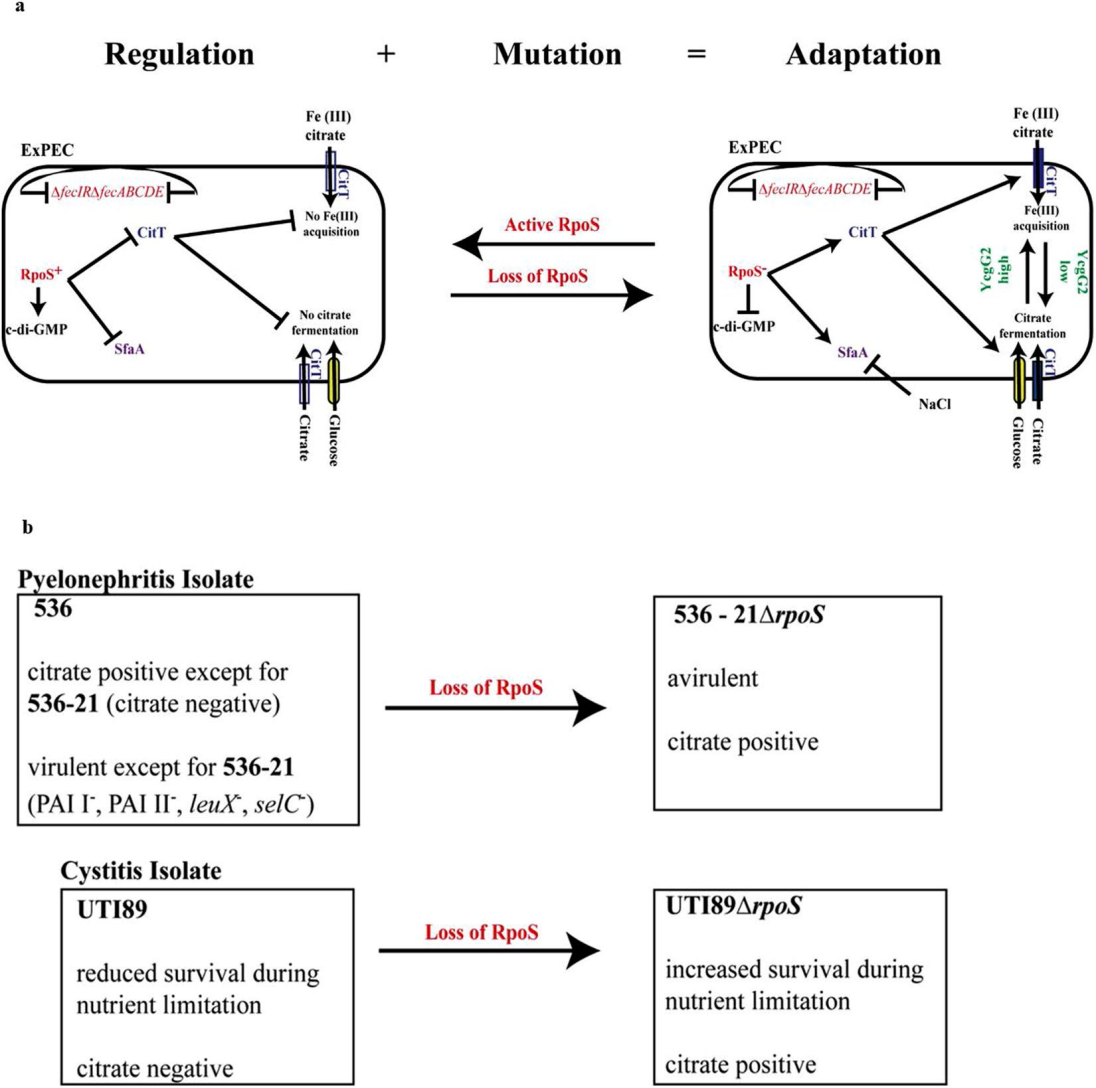
A model representing the pathoadaptive events in RpoS-deficient ExPEC strains. (**a**) Regulatory and mutational events define bacterial adaptation, exemplified by ExPEC and especially NMEC, which has naturally lost the RpoS regulator. Loss of RpoS leads to citrate utilization and ferric citrate uptake in nutrient-limited environments. RpoS deactivation (in red) leads to low c-di-GMP levels and triggers the production of CitT (in blue) and SfaA (in purple). Once CitT (in blue) is expressed, the bacteria can take in citrate and further ferment it, stimulated by low YcgG2 levels (in green). Low YcgG2 levels also lead to a decrease in SfaA (in purple) production. Due to the lack of *fecIRfecABCDE* (shown in red), the bacteria utilize CitT (in blue) to import ferrous iron citrate, whose utilization is further increased in the absence of YcgG2 (shown in green). These results are abolished when RpoS is restored. (**b**) The signalling events leading to metabolic novelty can be restored in UPEC. In the case of pyelonephritis isolates, citrate utilization is naturally occurring in the virulent 536 derivatives, while in the avirulent 536-21 strain, which is citrate-negative, utilization can be restored upon loss of RpoS. The UTI89 cystitis isolate does not utilize citrate, but utilization can be restored in the absence of RpoS, which also increases the survival of UTI89 in restrictive environments.

Retrieval of iron (III) from the niche of their host through the use of small molecules with high affinity to ferric ions (siderophores) is an important feature of commensal and pathogenic bacteria, including *E. coli*. In addition to serving as a carbon and energy source, another property of citrate is to form a complex with Fe^3+^ ions and thus be used by *E. coli* bacteria as another iron (III) acquisition system *via* a dedicated transport system encoded, together with its regulators, by the conserved *fecIRfecABCDE* gene cluster. Unexpectedly, we found that the genome of IHE3034 does not carry this gene cluster due to a large deletion event (Fig. 5b), which is also true for a large set of NMEC strains (Fig. S5a) and other ExPEC strains (including strains 536 and UTI89) (Fig. S5b). Before we tested if CitT could serve as an alternative importer of ferric ions in the form of ferric citrate complex, we detected that general iron (III) utilization by IHE3034 and its *∆citT*, *∆ycgG2* and *∆citT∆ycgG2* mutants was not altered, demonstrated by the formation of a yellow halo around each of the tested bacterial colonies in a CAS assay (Fig. 5c). Next, we challenged the bacteria with medium containing 1% Fe^3+^C(OH)(COO)_3_^3−^ in soft 3% agarose solution overlaid on LA. The wild-type IHE3034 bacteria managed to import the iron from the environment and to overload their cells with it, which resulted in a rust colour of their colonies (Fig. 5d; see Box I). On the other hand, *∆citT* mutant cells failed to extract iron from the medium and hence did not grow (Fig. 5d; see Box I). The *∆ycgG2* and *∆citT∆ycgG2* mutants also failed to grow in the medium, exhibiting the same colony phenotype as the IHE3034 *∆citT* mutant (Fig. 5d, Box II), which was also confirmed when the wild-type IHE3034 bacteria tested positive relative to the *∆ycgG2* mutant cells (Fig. 5d, Box III). Taken together, the data suggested the existence of a link between two metabolic CitT-dependent processes in which YcgG2 levels serve as a switch, *i.e*., high levels of YcgG2 trigger iron acquisition, while low levels trigger citrate fermentation (Fig. 6).

## Discussion

Selection for the adaptive mutations of commensal bacteria that settle in a new host niche often advance the shift from commensalism to pathogenicity - a shift that can be reversed if the expressed survival traits are induced in a controlled manner. In this study, we tested the ability of NMEC cells to grow and produce the SfaA major fimbrial subunit in the presence and absence of active RpoS. In general, the presence of RpoS activity increased 10-fold the cellular level of c-di-GMP and restored the formation of the rugose morphotype, as observed when bacteria were plated on CR plates, with equal efficiency in the wild-type IHE3034 background and in the background of its stand-alone PDE mutants (*∆sfaY* or *∆ycgG2*). Previous work by Wang and Kim showed that when RpoS activity was restored in IHE3034, the bacteria became more resistant to low pH (2.8), high osmolarity (4.8 M NaCl) and heat shock (54 °C)^29^. Therefore, we suggest that this could contribute to increased survival outside the host. However, the urinary bladder is a complex environment with buffered pH (6-7), a high concentration of NaCl (0.29 M NaCl maximum), and a dissolved oxygen level of 4 ppm^47–49^. Immunoblot experiments with bacterial samples from AUM showed that an environmental cue stimulated the synthesis of SfaA when IHE3034 bacteria were cultured in AUM, unlike LB, in which the expression of SfaA was moderate. SfaA expression was completely repressed when IHE3034 cells were cultured in 0.5 M NaCl. Whitman *et al*. showed that the expression of the *fim* and *foc* genes (coding for Type 1 and F1C fimbriae) is stimulated by urea but not by high concentrations of NaCl^50^. We found that *sfa* expression did not follow the same tendency: 0.5 M NaCl and 0.17 M urea decreased SfaA production, even though the *foc* and *sfa* genes have high homology and show the same regulatory trends^51^. S-fimbriation is a virulence feature shared between UPEC and NMEC strains, which provides them with adhesive properties towards sialylated glucoconjugates, *i.e*., S-fimbriae can bind to bladder and kidney epithelial cells to promote UPEC colonization (together with other virulence-associated fimbriae), and they also bind to erythrocytes and sialylated proteins of the brain tissue to promote the colonization of NMEC^17,52,53^. One of the main differences between UPEC and NMEC is the RpoS inactivation found in NMEC prototype strains^54^. Here, we showed that when RpoS activity was restored, the expression of S-fimbriae was downregulated, including in NMEC strains cultured in AUM. The fact that S-fimbrial expression is triggered in RpoS^−^ NMEC cells upon culture in AUM and repressed when RpoS activity is restored or when bacteria are cultured in 0.5 M NaCl provides NMEC with the opportunity to use maternal urine as a temporal residency before transmission from mother to child during delivery, which also suggests that NMEC could be an agent for neonatal urinary tract infections besides sepsis and meningitis.

Another example of pathoadaptive mutation is the remake of the *ycgG* gene (a stand-alone c-di-GMP PDE), which resulted in a new allele, *ycgG2*. Two groups, Sarenko *et al*. and Reinders *et al*., showed independently that YcgG expression levels were very low when bacteria were incubated under standard laboratory conditions, and the deletion mutant did not show any growth difference compared to that of the K-12 wild-type strain^55,56^. We also confirmed that YcgG2 exhibited conserved c-di-GMP phosphodiesterase activity, as evidenced by the intermediate rugose-smooth transition of *V. cholerae luxO*^*c*^. The *∆ycgG2* mutant showed decreased production of SfaA when the mutant cells were cultured in LB and AUM, which indicates that *sfa* gene regulation is c-di-GMP-dependent. When RpoS activity was restored in the deletion mutant, SfaA levels were further decreased, including when *∆ycgG2* cells were cultured in AUM. The importance of environmental conditions highlights the link between c-di-GMP signalling and the production of virulence factors. The expression of SfaA was optimal in AUM, while the expression of YcgG2 was decreased, suggesting that environmental stimuli have a dominant effect over c-di-GMP signalling and S-fimbrial biogenesis. Under all conditions, deletion of *ycgG2* led to reduced production of SfaA. Spurbeck *et al*. studied c-di-GMP signalling in the UPEC strain CFT073, in which the deletion in locus C1610 corresponds to the *ycgG2* allele, and the authors showed that this deletion led to an increase in the expression of FimA (major subunit of type 1 fimbriae) and to a decrease in the adherence to bladder epithelial cells^35^. This observation could be due to the downregulation of F1C-fimbriae in the absence of YcgG2, as in the case of strain IHE3034 and S-fimbriae.

Thomas Ferenci proposed a concept based on the existence of a conflict between adaptation to nutrient limitations and to stress resistance due to the competition of RpoS and RpoD for the core RNA polymerase complex, called SPANC (self-preservation and nutritional competence)^26,57^. His studies shed light on how the presence or absence of RpoS exerts pleiotropic effects on substrate utilization by certain bacteria; *E. coli* K-12 bacteria inactivate RpoS when challenged with glucose- or nitrogen-limiting sources, leading to activation of their nutrient-scavenging programmes, which comes with the loss of general cross-resistance^25^. Our findings revealed how the loss of RpoS may generate metabolic novelties regulated by YcgG2, *i.e*., mediated by changes in c-di-GMP levels. Here, we showed that neuroinvasive *E. coli* IHE3034 can utilize citrate aerobically due to RpoS deficiency and c-di-GMP regulation when a co-substrate (glucose) is provided. In addition, citrate is taken in by the CitT antiporter, which is also aerobically expressed. Fermentation of citrate is further accelerated by the absence of either of the two stand-alone c-di-GMP PDEs, SfaY or YcgG2, which suggests that this process is predominantly used by non-motile bacteria. Citrate utilization by NMEC strains is an important metabolic upgrade when their cells are in the gut or challenged in environments poor in oxygen and carbon and energy sources. This would presumably be the case in urine, in which the glucose concentration can vary between 0.05 and 3.4 mM^58,59^, the citrate concentration between 1–4 mM^60^ and in which the oxygen saturation is 4 ppm^49^. Snyder *et al*. showed that when UPEC bacteria thrive in the urinary tract, they are moderately aerobic with a preference for amino acids and peptides as carbon and energy sources^61^. The new metabolic pathway described here, triggered by the lack of RpoS, could provide bacteria with an upgrade allowing the use of more substrates, such as citrate and glucose. The fact that citrate utilization could be restored in the cystitis isolate showed the potential of this strain to trigger the programme upon loss of RpoS when needed. Moreover, the vast majority of the tested pyelonephritis isolates could already use citrate, which suggests that this upgrade is already present not only in NMEC but in pyelonephritis isolates with previously demonstrated virulence properties.

In addition to serving as a carbon and energy source, citrate can also serve as a chelator agent of Fe(III). When it forms complexes with Fe(III), it can be internalized by the FecA/B system to the bacterial periplasmic space^62,63^. Since IHE3034 (and the majority of ExPEC strains) does not have the FecA/B system, we suggest that IHE3034 bacteria use the CitT antiporter for ferric citrate uptake - an ability that ceases when YcgG2 is not present. Iron citrate is mostly available in the gut, so it is interesting to speculate that during the commensal phase of their lifestyle, IHE3034 bacteria express YcgG2, which triggers iron (III) citrate uptake since many carbon sources are available and there is no need for citrate fermentation. Once they escape the gut, the IHE3034 cells reduce the production of YcgG2 (as shown here when they are grown in artificial urine) and trigger the citrate fermentation programme, since carbon sources become restricted and iron uptake is performed by siderophores. Thus, YcgG2 could serve as a switch, i.e., high levels trigger iron (III) uptake, while low levels trigger citrate fermentation. CitT is a citrate/succinate antiporter^4^, and one plausible mechanism for the switch could be the reduction in an exported negatively charged molecule, such as succinate, due to reduced citrate fermentation when YcgG2 is present. Hypothetically, this could allow another exported molecule(s) to complete the transfer, bringing iron citrate complex into the cell instead of citrate. Along with its role in citrate fermentation and Fe(III) uptake, deletion of *ycgG2* reduced the production of SfaA (the major structural protein of the S-fimbriae), which broadens its role as a regulator that participates in the bacterial transition from commensal to pathogen and *vice versa*.

C-di-GMP signalling plays a major role in bacterial colonization but, in the case of NMEC, also provides the bacteria with the opportunity to benefit from available environmental carbon, energy and iron sources - an opportunity that would not be possible in the presence of active RpoS. Environmental cues combined with evolved regulatory node(s) could serve as a trigger for a reversible commensal-pathogen transition, defined by the levels of YcgG2, a c-di-GMP phosphodiesterase, in the absence of the global stress regulator RpoS.

## Methods

### Media and growth conditions of bacterial strains

All bacterial strains (listed in Table S3) were cultured aerobically at 37 °C, unless stated otherwise in a particular section. Cells were cultured in plain lysogenic broth (LB) or agar (LA), LB supplemented with 0.5 M NaCl or in artificial urine medium (AUM)^64^ with the appropriate amount of antibiotic(s) or inducer when needed: carbenicillin −100 µg/ml; chloramphenicol −25 µg/ml; L-arabinose −1 mM. Congo Red (CR) agar supplemented with 40 µg/ml Congo Red dye, and 20 µg/ml Coomassie Brilliant Blue R-250 dye (with the appropriate amount of antibiotic(s) or inducer when needed) was used for smooth-rugose screening and macromorphological colony analysis. Columbian agar supplemented with 5% horse erythrocytes was used to assess the haemolytic properties of the Finnish pyelonephritis isolates.

### Genetic experiments

All plasmids and primers used in this work are listed in Tables S4 and S5, respectively. The sequences of the *E. coli* IHE3034 (accession number: NC_017628) and *E. coli* MG1655 (accession number: NC_000913) genomes were used to perform all genetic experiments in this study. The molecular genetic experiments were performed as described by Sambrook *et al*.^65^.

### Construction of deletion mutants

*E. coli* IHE3034*∆ycgG2* was constructed with the help of a lambda-red recombinase system^66^, while *E. coli* IHE3034*∆sfaY* was created in a scarless manner with the help of the suicide pKO3 plasmid^67^. In-frame deletion of *citT* and *rpoS* was introduced *via* P1 transduction^68^.

### Construction of the *E. coli* IHE3034*∆ycgG2::cat* mutant strain

The deletion fragment was generated *via* PCR with the *ycgG2*delFw and *ycgG2*delRv primers, using the pKD3 plasmid as a template. IHE3034/pSIM6 electrocompetent cells with the induced lambda-red recombinase system encoded on the pSIM6 plasmid were electroporated (2500 V, 200 Ω, 25 μF) with 1 µg of the *∆ycgG2* fragment and were allowed to recover overnight at 37 °C aerobically^69^. The day after, cells were plated on LA/Cm^25^, and after an overnight incubation, a few colonies were checked for proper allelic replacement *via* sequencing of the *ycgG2* mutated region with the 1312Fw and *ycgG2*dwdelRv primers.

### Construction of the *E. coli* IHE3034*∆sfaY* mutant strain

The *sfaY* gene was targeted by a deletion fragment cloned into the suicide pKO3 plasmid. The deletion fragment was generated *via* crossover PCR using chromosomal IHE3034 DNA as a template. The first PCR with the EAL6up and EALdelendup primers created the upstream deletion fragment with 304-bp homology to the target gene and its upstream region, while the 304-bp downstream deletion fragment was created with primers EALdelstartdo and SfaII-3. The two deletion fragments shared 24 bp of complementarity, introduced by the EALdelendup and EALstartdo primers, which were used in the second PCR in which the two deletion fragments were used as templates that, with the help of the EAL6up and SfaII-3 primers after amplification, produced the full *∆sfaY* deletion fragment with introduced *EcoR*I (at the 5′) and *Sal*I (at the 3′) restriction sites. The generated deletion product was digested with *EcoR*I and *Sal*I and cloned into pKO3. The construct was electroporated into the IHE3034 cells, and after the creation of the heterogenotes, subsequent mutagenesis steps were performed as described by Link *et al*.^67^. The obtained mutant strain was verified *via* sequencing using the Fw*sfaY*dw and dd*sfaX*Rv primers.

### Construction of *citT* and *rpoS* in-frame deletion mutants *via* P1 transduction

*E. coli* JW0604-1*∆citT750::kan* was used as a donor of *citT* deletion and *E. coli* RH90 as a donor for the *∆rpoS*::Tn10 deletion fragment. P1 bacteriophages, unable to lysogenize, were recycled twice on strain JW0604-1 or RH90 by the confluent plate lysis technique. The obtained P1[JW0604-1*∆citT750::kan*] viral lysate was used for transducing the target strains IHE3034 and IHE3034*∆ycgG2*, and P1[RH90*∆rpoS*::Tn10] was used for target strains UTI89, 536-21 and 536R3. For 1 transduction reaction, 0.5 ml of 10^9^ target bacteria in their exponential phase of growth were mixed with 0.5 ml of P1 lysate and 0.5 ml of P1 solution (0.015 M CaCl_2_ and 0.03 M MgSO_4_). Transduction took place for 20 min at 37 °C. Afterwards, cells were spun down, and after washing with saline, the cells were plated on selective antibiotic agar plates. To verify that the deletion of *citT* and *rpoS via* allelic replacement took place and to exclude any polar effects, the mutated alleles were PCR-amplified and sequenced with the primers up*citT*Fw and dw*citT*Rv for *∆citT750::kan* and the primers 3120FW and *rpoS*Rv for *∆rpoS::Tn10*.

### Cloning of *ycgG* and *ycgG2* into the pBAD18 vector

The *ycgG* (GeneID: 945738) and *ycgG2* (GeneID: 12689880) genes, together with their native RBS sequences, were cloned into the pBAD18 plasmid (accession number: X81838.1) downstream of the P_BAD_ promoter in a 5′- > 3′ orientation, which allowed the promoter to regulate the expression of the cloned genes. The *ycgG* gene was amplified *via* PCR using the genomic DNA of MG1655 with the *ycgG*K-12Fw and *ycgG*K-12Rv primers, which also introduced the *Sac*I and *Hind*III restriction sites into the 5′ and 3′ ends, respectively. After double restriction digestion of the gene fragments and the plasmid, the genes were cloned into the vector. The positive clones were verified *via* sequencing of the constructs with pBAD18Fw and pBAD18Rv primers.

### Whole genome sequencing of *E. coli* IHE3034F

The chromosomal DNA of IHE3034F was extracted and subjected to next generation-sequencing (NGS). For NGS of the IHE3034F chromosome, we used the services of Admera Health, LCC. In short, the DNA library was prepared using the Illumina Nextera XT library prep kit and then run on an Illumina MiSeq Nano PE 150 Cycle, giving 60X coverage and a read length of 2 × 150 (paired end). The reads were directly mapped to the IHE3034 reference genome by using BWA mem (version: 0.7.17) with the default parameters. The variants were called by using GATK HaplotypeCaller (version 4.0.2.1) (-stand-call-conf 30 -ploidy 1, other parameters are default). Filtering for low-quality calls was performed with GATK VariantFiltration for SNPs and indels separately (SNPs filtering criteria: QD < 2.0 || FS > 60.0 || MQ < 40.0; indel filtering criteria: QD < 2.0 || FS > 200.0).

## Biochemical Experiments

### Western immunoblotting

Overnight cultures of bacteria were pelleted by centrifugation and resuspended in 20 mM TRIS-HCl and 4X Laemmli sample buffer. Equal amounts from each sample were electrophoresed (SDS-PAGE, 15% polyacrylamide/bis-acrylamide concentration) and then transferred onto a polyvinylidene fluoride (PVDF) microporous membrane To detect the levels of YcgG2, a monoclonal antibody against the C-terminal part of the full-length YcgG (Abmart, X-P75995-C) was used, since the C-terminal part remains coded. To detect SfaA, a polyclonal serum against purified S-fimbriae, a kind gift from Prof. Dr. Jörg Hacker, was used. To detect RpoS, a monoclonal antibody against RpoS (NeoClone, WP009) was used. YcgG2 *in vitro*-produced protein was used as a positive control, while the levels of OmpA or H-NS were used as a loading control, detected by rabbit polyclonal anti-H-NS^70^ or anti-OmpA^71^ antisera. Protein bands were visualized using the ECL method described by the manufacturer (GE Healthcare Life Sciences) and detected using the LAS4000 Image Quant system, GE Healthcare Life Sciences. One representative replicate of three independent repetitions is shown.

### *In vitro* transcription/translation of the YcgG and YcgG2 proteins

PURE*frex* 2.0 is a system that contains all obligatory *E. coli* factors required for gene expression up to a fully synthesized protein (GeneFrontier Corporation) when a certain template is present. Linearized templates that carried the *ycgG* and *ycgG2* alleles individually under the regulation of the T7 RNA polymerase promoter were generated. Six nanograms of each template was used in a PURE*frex* 2.0 reaction for the generation of protein species, according to the manufacturer’s protocol (GeneFrontier Corporation). For radioactive labelling, 2 µl of [35-S]-L-cysteine and [35-S]-L-methionine (EasyTag™ EXPRESS^35^S Protein Labeling Mix, 2 mCi) was added into the PURE*frex* 2.0 mixtures. After incubation, the samples were run on SDS-PAGE, and gels were dried and exposed overnight in an imager cassette. The screens of the cassettes were then scanned by a Typhoon FLA 9500 laser scanner (GE Healthcare Life Sciences). One representative replicate of three independent repetitions is shown.

### Protein identification

For the identification of YcgG2, we used the services of Proteome Factory (Berlin, Germany). In short, the presence of the *ycgG2* gene product and its size were confirmed *via in vitro* transcription/translation followed by digestion with Glu-C endopeptidase. The generated peptides were detected *via* ESI-Ion TRAP mass spectrometry.

### C-di-GMP measurements

The total amount of cyclic nucleotides was extracted from approximately 1.6 × 10^10^ bacteria grown aerobically on LA at 37 °C  overnight according to a previously described method^72^. Measurements were performed with biological triplicates and presented as molar concentrations.

### Phenotypic experiments

#### Rugose-Smooth screening assay

*Vibrio cholerae luxO*^*c*^ – derived strains NZ44, NZ92 and NZ93 were cultured in LB/Cb^100^ overnight, and then 10 µl from each culture was spotted on CR plates (with 1 mM L-arabinose and Cb^100^). The plates were incubated at 37 °C for 48 hours. In the presence of c-di-GMP phosphodiesterase activity, a transition from rugose (wrinkled) to a smooth colony appearance was observed. One representative of three independent experiments is shown.

#### Macrocolony formation assay

Five microlitres of overnight *E. coli* culture was spotted on CR plates and incubated for 5 days at different temperatures: 22 °C, 30 °C and 37 °C. Macrocolonies were evaluated according to their change in colony appearance, *i.e*., a transition from the smooth to the rugose morphotype. One representative of three independent experiments is shown.

#### Citrate utilization assay

Five microlitres of different serially diluted *E. coli* strains were spotted on Simmon’s citrate agar, embedded with 150 µl or 75 µl of 40% D-glucose, referred to later in the text as 0.2% or 0.1%, respectively. Bacteria were incubated aerobically at 37 °C or 22 °C for 5 to 10 days. The glucose was embedded to form a gradient as a co-substrate providing the reducing power required for citrate utilization^3^. If a blue halo was present around the colonies, growth on citrate was considered positive. If the halo was yellow, bacteria were able to utilize only the glucose and were unable to feed on citrate. One representative of three independent experiments is shown.

#### Blue agar chrome azurol S (CAS) assay for siderophore detection

The assay was performed as described by Schwyn and Neilands with a few modifications^73^. To prepare the blue dye, 0.06 g of chrome azurol S was dissolved in 50 ml of water and mixed with 9 ml of solution containing 0.0027 g of FeCl_3_X6H_2_O dissolved in 10 ml of 10 mM HCl. Finally, the solution was mixed with 40 ml of solution containing 0.073 g of HDTMA (hexadecyltrimethylammonium bromide). One hundred millilitres of the blue dye was added to 900 ml of melted 1.1XLA and poured on plates. Incubation of the bacteria was performed overnight at 37 °C in triplicate. One representative of three independent experiments is shown.

#### Ferric citrate utilization assay

A 1% aqueous solution of Fe(III) citrate was used as the dissolvent for 3% agarose. After boiling, the semisolid Fe(III) citrate agarose was overlaid on LA plates to create a biphasic environment. One hundred microlitres of overnight bacterial culture was injected into the agarose and incubated overnight at 37 °C. If bacteria were able to extract and use the iron chelated by the citrate, their appearance became brown. One representative of three independent experiments is shown.

### Statistical Analysis

Statistical analysis of the experiments was performed with GraphPad Prism 7.04. Multiple comparisons of samples was carried out with one-way ANOVA with Bonferroni’s correction, and t-tests were performed for two-sample comparisons. Significance was determined by a P-value < 0.05, as follows: P > 0.05 (ns, *i.e*., not significant); P ≤ 0.05 (*); P ≤ 0.01 (**); P ≤ 0.001 (***).

### Bioinformatics

DNA sequences from the genomes of various ExPEC and commensal strains were used for multiple alignments with the following accession numbers: IHE3034 (NC_017628.1), RS218 (CP007149.1), S56 (NZ_CP010242.1), S50 (NZ_CP010238.1), S42 (NZ_CP010236.1), S40 (NZ_CP010235.1), S30 (NZ_CP010231.1), S21 (NZ_CP010230.1), S10 (NZ_CP010229.1), S1 (NZ_CP010226.1), NMEC O18 (NZ_CP007275.1), S43 (CP010237.1), S3 (CP010228.1), NA114 (CP002797), UTI89 (CP000243), APEC O1 (NC_008563.1), CFT073 (NC_004431.1), 536 (NC_008253), UPEC 26-1 (NZ_CP016497), K-15KW01 (CP016358), NGF1 (NZ_CP016007), ECONIH2 (NZ_CP014667), SF-173 (NZ_CP012631), Nissle 1917 (CP007799.1), Di2 (CP002211.1), UM146 (CP002167.1), ABU83972 (CP001671.1) and MG1655 (NC_000913). For multiple sequence global alignments, Clustal Omega software was used, while pairwise sequence alignments were performed with EMBOSS Needle. SnapGene software (from GSL Biotech; available at snapgene.com) was used for the graphical representation of multiple global alignments.

## Supplementary information

Absence of Global Stress Regulation in Escherichia coli Promotes Pathoadaptation and Novel c-di-GMP-dependent Metabolic Capability
