# Supplementary material for: Absence of Global Stress Regulation in Escherichia coli Promotes Pathoadaptation and Novel c-di-GMP-dependent Metabolic Capability
Source: Sci Rep. 2019 Feb 22;9:2600. doi: 10.1038/s41598-019-39580-w (PMC6385356; doi:10.1038/s41598-019-39580-w)
Supplement: Supplementary file 1 — Absence of Global Stress Regulation in Escherichia coli Promotes Pathoadaptation and Novel c-di-GMP-dependent Metabolic Capability [file 41598_2019_39580_MOESM1_ESM.pdf]

Absence of Global Stress Regulation in *Escherichia coli* Promotes Pathoadaptation and Novel c-di-GMP-dependent Metabolic Capability

Nikola Zlatkov[1], Bernt Eric Uhlin[1*]

[1]The Laboratory for Molecular Infection Medicine Sweden (MIMS), Department of Molecular Biology, Umeå University, Umeå, Sweden

[*]Corresponding author: **bernt.eric.uhlin@umu.se**

a

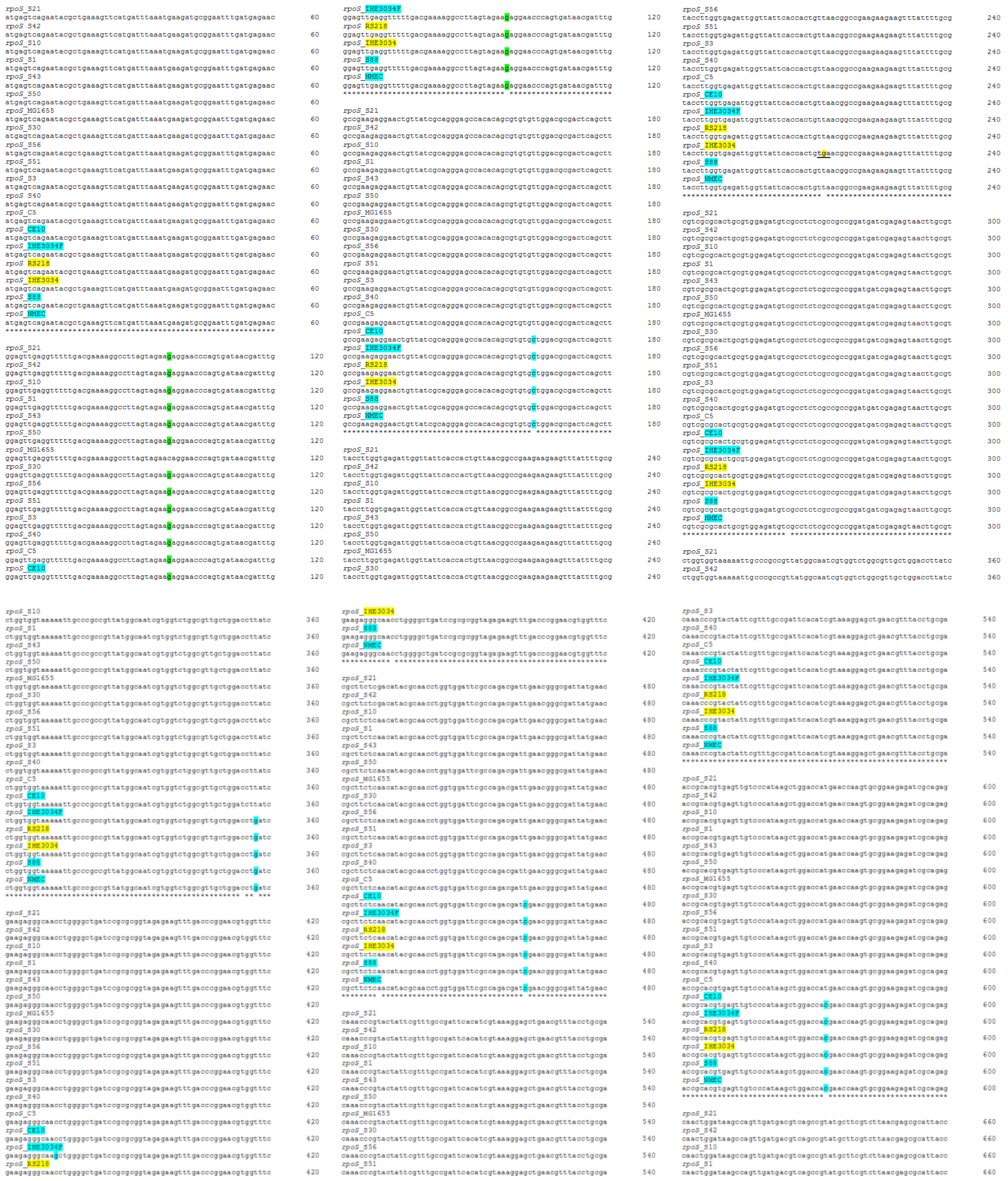

Fig. S1

a (extended)

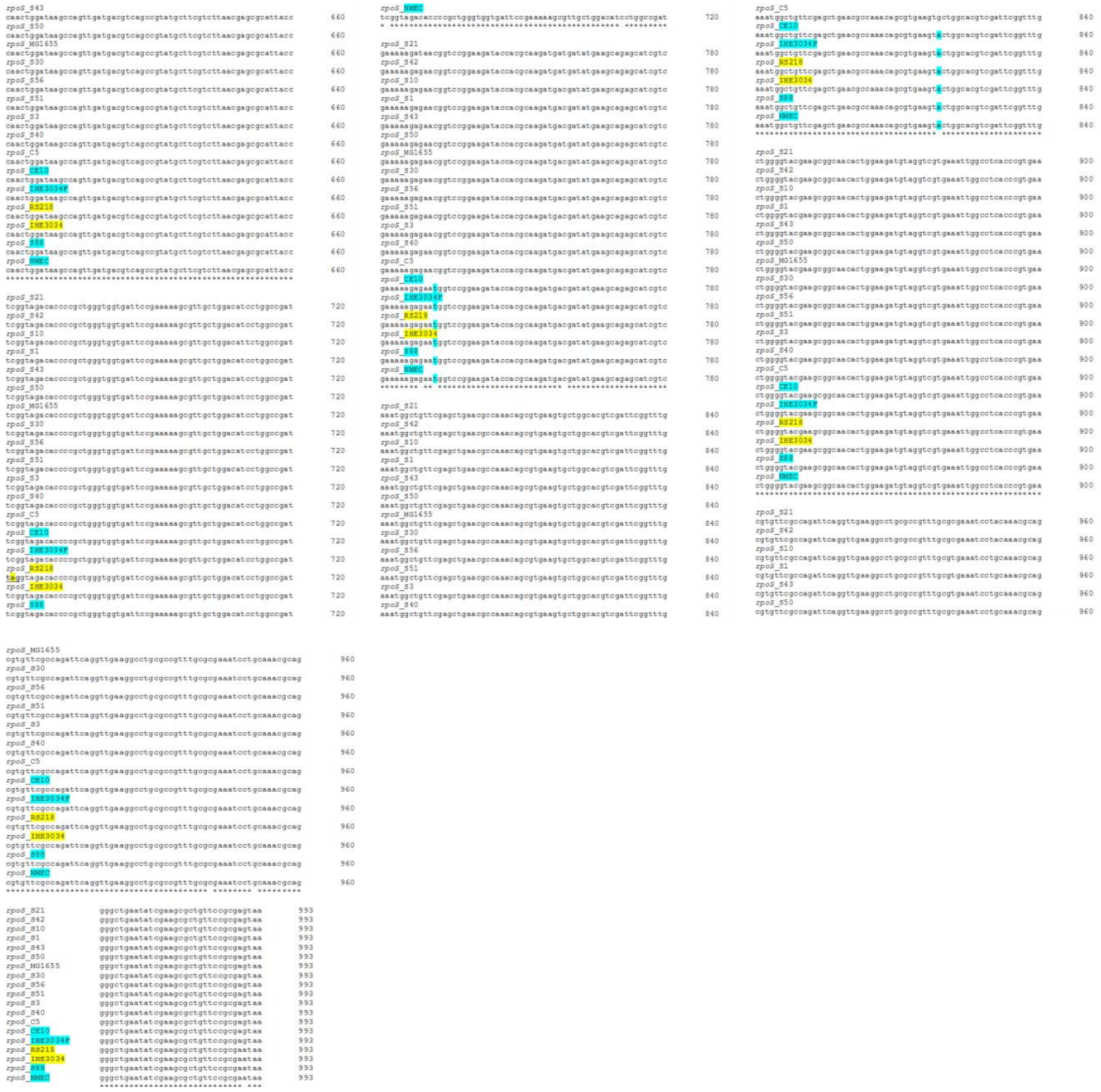

Fig. S1

b

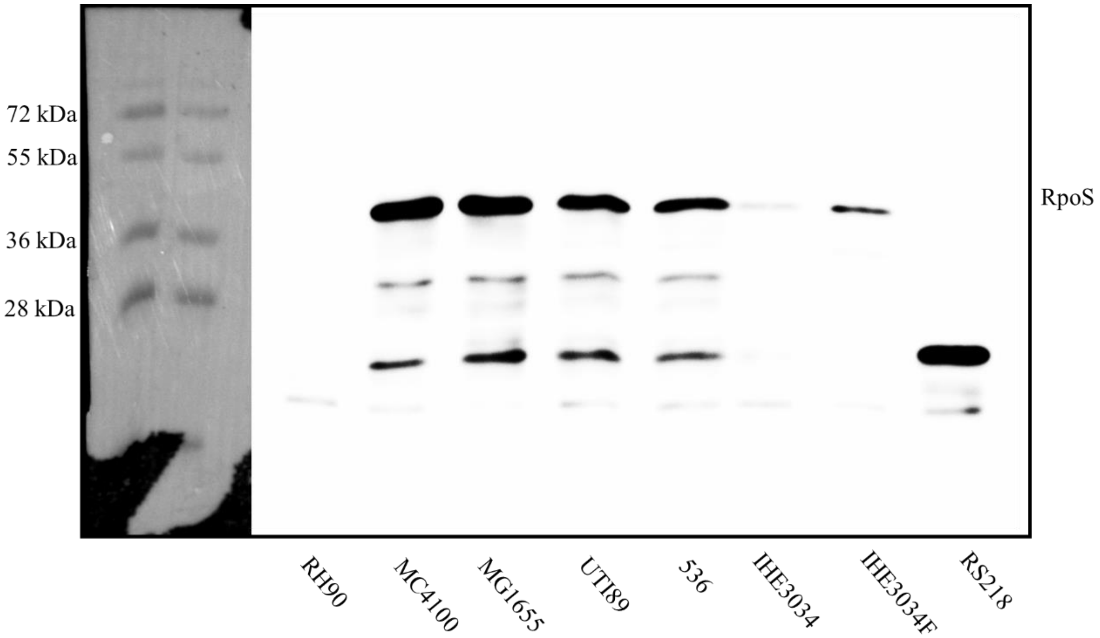
72 kDa
55 kDa
36 kDa
28 kDa
RpoS
RH90
MC4100
MG1655
UTI89
536
IHE3034
IHE3034F
RS218

c

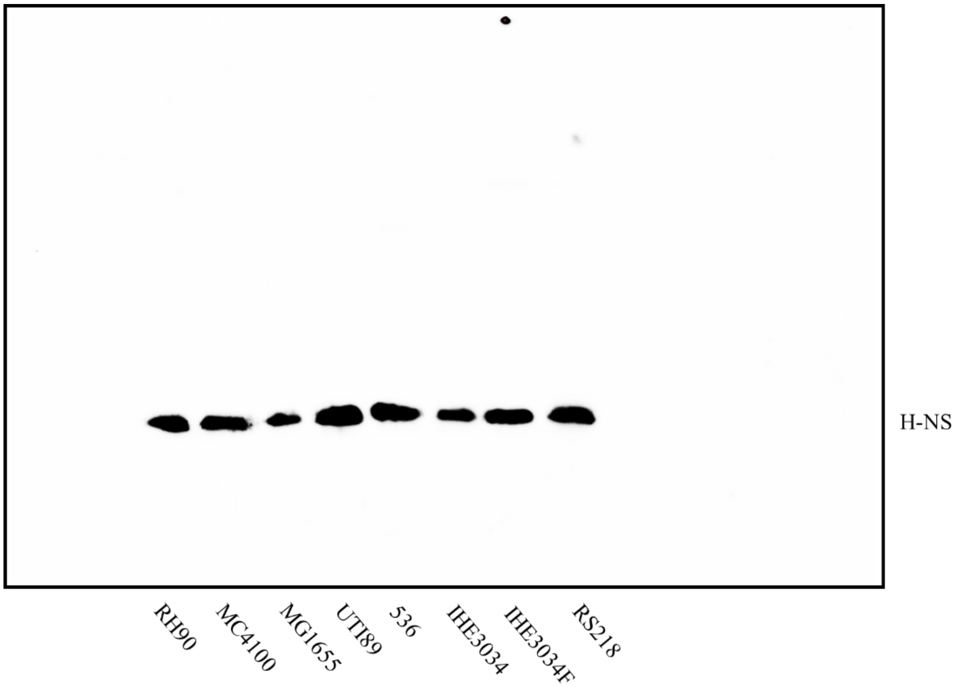
H-NS
RH90
MC4100
MG1655
UTI89
536
IHE3034
IHE3034F
RS218

Fig. S1

d

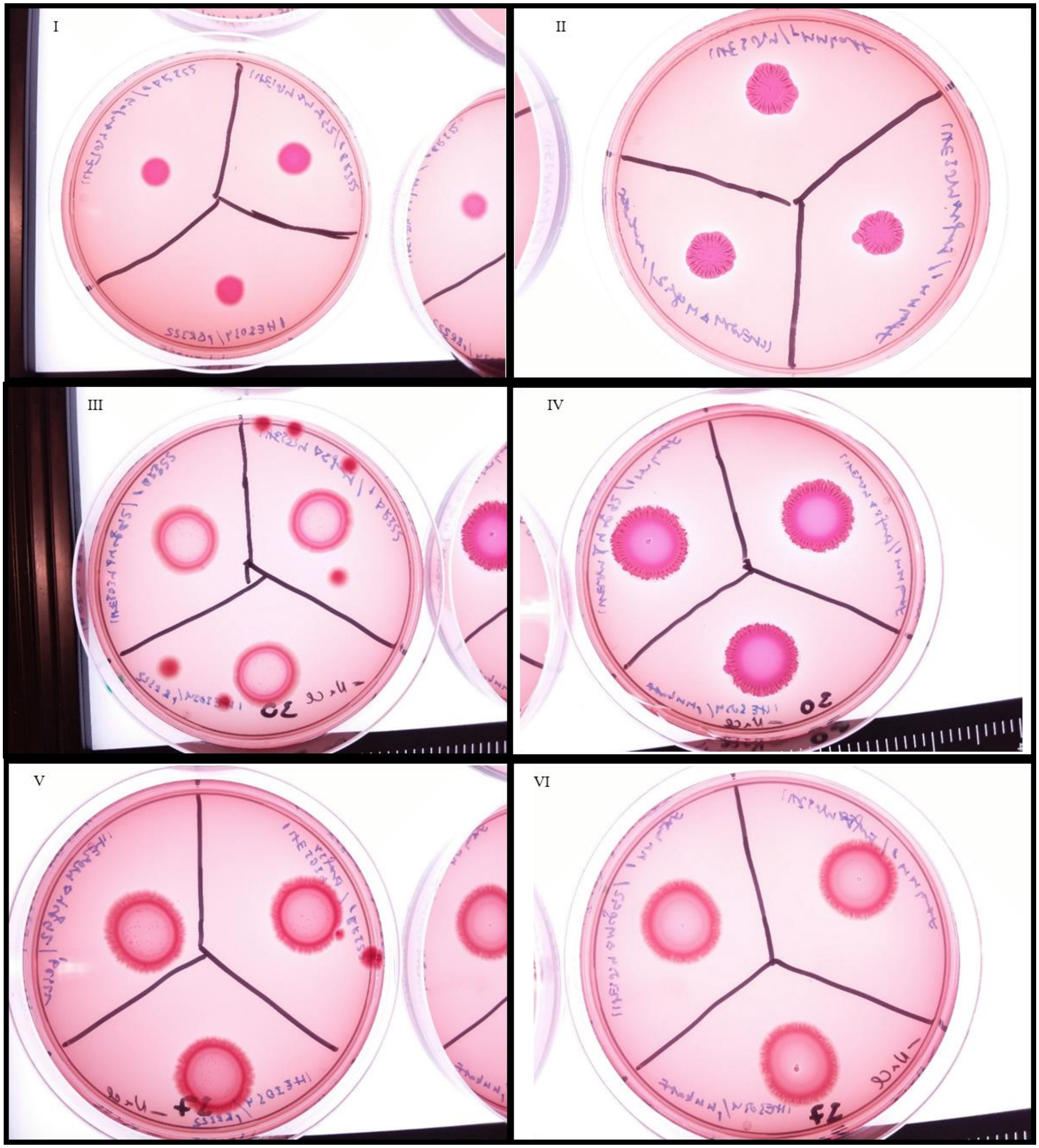

Fig. S1: Properties of the *rpoS* allele in IHE3034.

(a) Multiple sequence alignment of the *rpoS* allelic variants of NMEC compared to the *rpoS* allele of MG1655. Highlighted in yellow are the strains in which *rpoS* gene is turned into a pseudogene, the point mutation that introduced the stop codon shown in bold and underlined. Highlighted in blue are the point mutations that are typical for the *rpoS* allele of IHE3034F which expresses inactive RpoS.

The strains whose *rpoS* allele share the same point mutations of IHE3034F *rpoS* are highlighted in blue too. Common point mutations for all NMEC strains are highlighted in green.

(b) The full membrane of the immunoblot against RpoS present in **Fig.1c**.

(c) The full membrane of the immunoblot against H-NS present in **Fig.1c**.

(d) The full pictures of the Congo Red plates used for the smooth-rugose assay present in **Fig. 1d**.

a

>*ycgG2*_K1_IHE3034

ttg-ata-ttt-att-ctg-tta-atc-gcc-tgc-gcc-gct-gcc-ttc-ctg-ctt-gat-agg-tat-ttc-aat-aaa-agc-gca-acg-cct-gaa-gag-atc-ctg-
cga-cgg-gct-ata-aat-aat-ggg-gag-atc-gtc-cct-ttt-tac-caa-cct-gtg-gta-aat-ggt-cgg-gaa-ggg-aca-ttg-cgg-gga-gtt-gag-
gtg-tta-gcc-cgc-tgg-aaa-caa-cct-cac-ggt-gga-tat-ata-tca-ccc-gcg-gca-ttt-att-cca-ctt-gct-gaa-aaa-tgc-gga-tta-atc-gtt-
ccg-ctt-acg-caa-agc-ctg-atg-aat-cag-gtt-gcc-aga-cag-atg-aac-gct-atc-gcg-agt-aaa-ctg-ccg-gaa-ggt-ttt-cat-att-ggg-
att-aat-ttt-agc-gcc-tcg-cat-att-att-tcg-ccg-acg-ttt-gtc-gac-gag-tgc-tta-aat-tac-cgt-gac-agt-ttt-acc-cgc-cgc-gat-tta-aac-
ctt-gtt-ctg-gaa-gtc-acc-gag-cgt-gag-cca-tta-aat-gtt-gat-gaa-agt-ctg-gtt-cag-cgg-ttg-aac-att-ctg-cat-gaa-aat-ggt-ttt-
gtc-atc-gcg-ctg-gat-gat-ttc-ggt-act-ggt-tac-tca-ggg-ctt-tct-tat-ctg-cat-gac-ttg-cat-att-gat-tat-atc-aaa-att-gat-cat-agt-
ttc-gtt-ggc-cgc-gtc-aac-gca-gac-cca-gca-tca-acc-cga-att-ctg-gat-tgt-gta-ttg-gat-ctg-gcg-cgt-aaa-ctt-tcg-atc-agt-atc-
gtc-gct-gaa-gat-gtc-gaa-acg-aaa-gaa-caa-ttt-gac-tat-ctg-aac-caa-aat-aat-atc-aca-ttt-cag-cag-ggt-tat-tat-ttc-tat-aaa-
cct-gtt-aca-tac-atc-gac-ctg-gtc-aag-att-atc-ctt-tct-aaa-ccg-aag-gtg-aag-att-gtg-gtt-gag-tga

b

```
YcgG        225 LIFILLIACAAAFLLDRYFNKSATPEEILRRAINNGEIVPFYQPVVNGRE   274
                :|||||||||||||||||||||||||||||||||||||||||||||||||
YcgG2         1 MIFILLIACAAAFLLDRYFNKSATPEEILRRAINNGEIVPFYQPVVNGRE    50

YcgG        275 GTLRGVEVLARWKQPHGGYISPAAFIPLAEKSGLIVPLTQSLMNQVARQM   324
                |||||||||||||||||||||||||||||||.||||||||||||||||||
YcgG2        51 GTLRGVEVLARWKQPHGGYISPAAFIPLAEKCGLIVPLTQSLMNQVARQM   100

YcgG        325 NAIASKLPEGFHIGINFSASHIISPTFVDECLNFRDSFTRRDLNLVLEVT   374
                |||||||||||||||||||||||||||||||||:||||||||||||||||
YcgG2       101 NAIASKLPEGFHIGINFSASHIISPTFVDECLNYRDSFTRRDLNLVLEVT   150

YcgG        375 EREPLNVDESLVQRLNILHENGFVIALDDFGTGYSGLSYLHDLHIDYIKI   424
                ||||||||||||||||||||||||||||||||||||||||||||||||||
YcgG2       151 EREPLNVDESLVQRLNILHENGFVIALDDFGTGYSGLSYLHDLHIDYIKI   200

YcgG        425 DHSFVGRVNADPESTRILDCVLDLARKLSISIVAEGVETKEQLDYLNQNY   474
                ||||||||||||.|||||||||||||||||||||||.||||||.||||||.
YcgG2       201 DHSFVGRVNADPASTRILDCVLDLARKLSISIVAEDVETKEQFDYLNQNN   250

YcgG        475 ITFQQGYYFYKPVTYIDLVKIILSKPKVKVVVE    507
                |||||||||||||||||||||||||||||:|||
YcgG2       251 ITFQQGYYFYKPVTYIDLVKIILSKPKVKIVVE    283
```

c

```
YcgG2        93 MNQVARQMNAIASKLPEGFHIGINFSASHIISPTFVDECLNYRDSFTRRD   142
                ||||||||||||||||||||||||||||||||||||||||||||||||||
YcgG2_ATG     1 MNQVARQMNAIASKLPEGFHIGINFSASHIISPTFVDECLNYRDSFTRRD    50

YcgG2       143 LNLVLEVTEREPLNVDESLVQRLNILHENGFVIALDDFGTGYSGLSYLHD   192
                ||||||||||||||||||||||||||||||||||||||||||||||||||
YcgG2_ATG    51 LNLVLEVTEREPLNVDESLVQRLNILHENGFVIALDDFGTGYSGLSYLHD   100

YcgG2       193 LHIDYIKIDHSFVGRVNADPASTRILDCVLDLARKLSISIVAEDVETKEQ   242
                ||||||||||||||||||||||||||||||||||||||||||||||||||
YcgG2_ATG   101 LHIDYIKIDHSFVGRVNADPASTRILDCVLDLARKLSISIVAEDVETKEQ   150

YcgG2       243 FDYLNQNNITFQQGYYFYKPVTYIDLVKIILSKPKVKIVVE    283
                |||||||||||||||||||||||||||||||||||||||||
YcgG2_ATG   151 FDYLNQNNITFQQGYYFYKPVTYIDLVKIILSKPKVKIVVE    191
```

d

```
YcgG        317 MNQVARQMNAIASKLPEGFHIGINFSASHIISPTFVDECLNFRDSFTRRD   366
                |||||||||||||||||||||||||||||||||||||||||:||||||||
YcgG2_ATG     1 MNQVARQMNAIASKLPEGFHIGINFSASHIISPTFVDECLNYRDSFTRRD    50

YcgG        367 LNLVLEVTEREPLNVDESLVQRLNILHENGFVIALDDFGTGYSGLSYLHD   416
                ||||||||||||||||||||||||||||||||||||||||||||||||||
YcgG2_ATG    51 LNLVLEVTEREPLNVDESLVQRLNILHENGFVIALDDFGTGYSGLSYLHD   100

YcgG        417 LHIDYIKIDHSFVGRVNADPESTRILDCVLDLARKLSISIVAEGVETKEQ   466
                ||||||||||||||||||||.||||||||||||||||||||||.||||||
YcgG2_ATG   101 LHIDYIKIDHSFVGRVNADPASTRILDCVLDLARKLSISIVAEDVETKEQ   150

YcgG        467 LDYLNQNYITFQQGYYFYKPVTYIDLVKIILSKPKVKVVVE    507
                .||||||.|||||||||||||||||||||||||||||:|||
YcgG2_ATG   151 FDYLNQNNITFQQGYYFYKPVTYIDLVKIILSKPKVKIVVE    191
```

Fig. S2.

e

>YcgG2_K1_IHE3034
MNQVARQMNAIASKLPEGFHIGINFSASHIISPTFVDECLNYRDSFTRRDLNLVLEVTEREPLNVDES
LVQRLNILHENGFVIALDDFGTGYSGLSYLHDLHIDYIKIDHSFVGRVNADPASTRILDCVLDLARK
LSISIVAEDVETKEQFDYLNQNNITFQQGYYFYKPVTYIDLVKIILSKPKVKIVVE

f

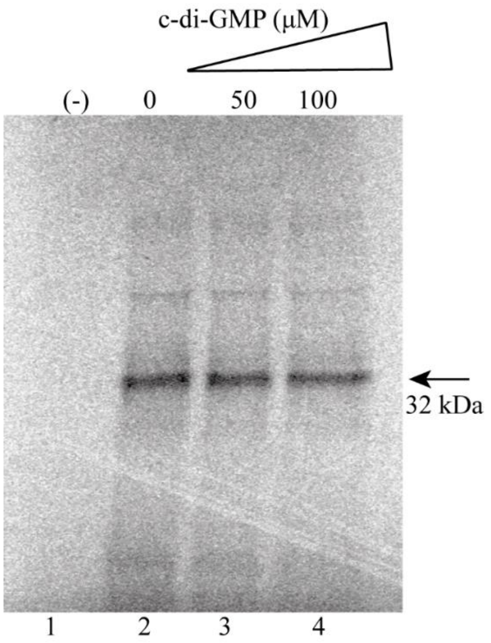

g

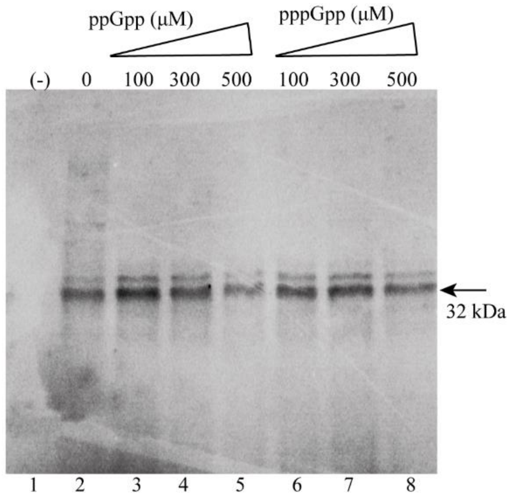

Fig. S2.

h

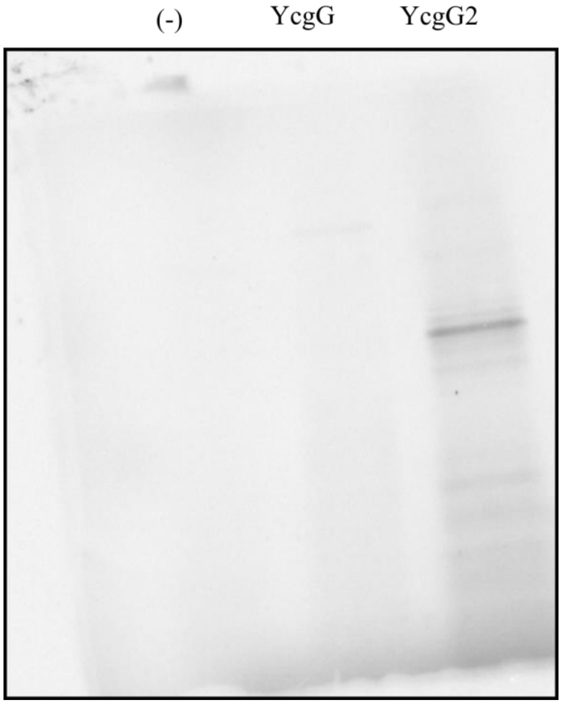

i

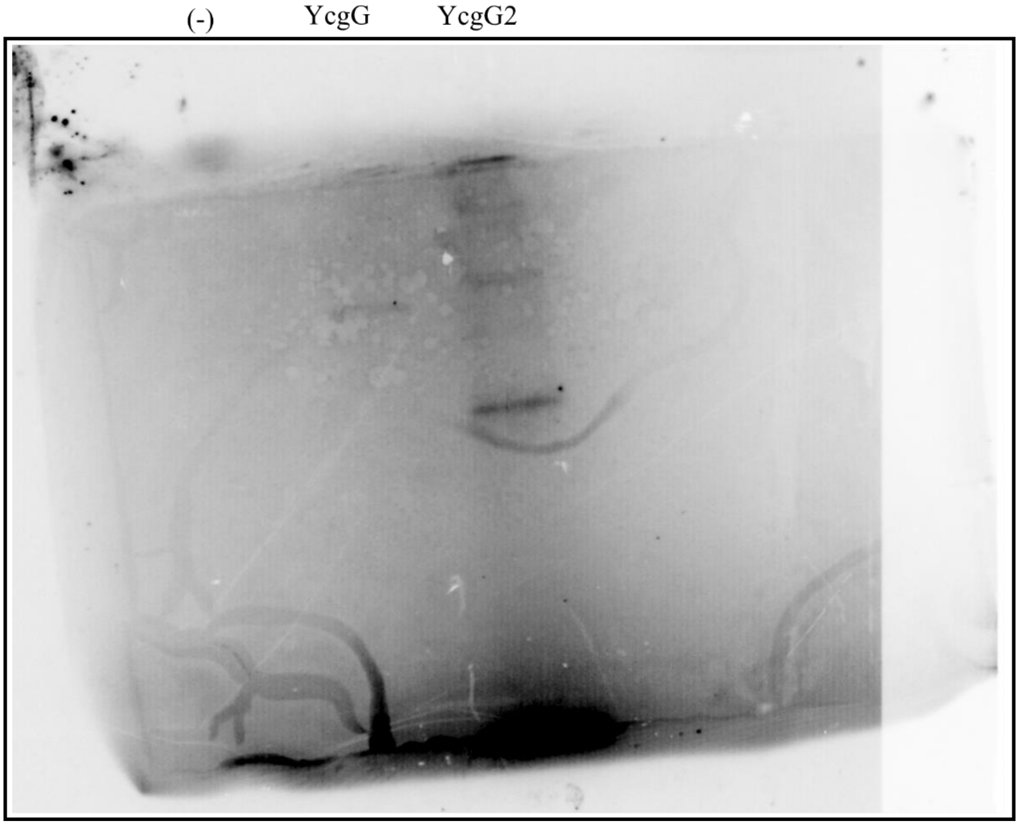

Fig. S2.

j

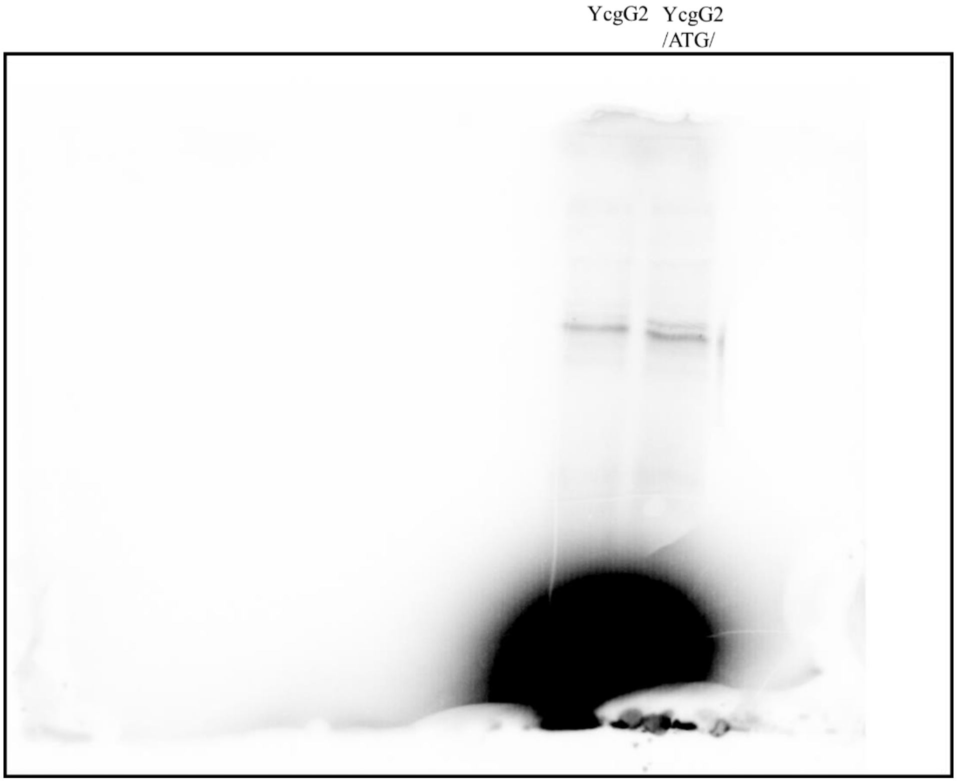

k

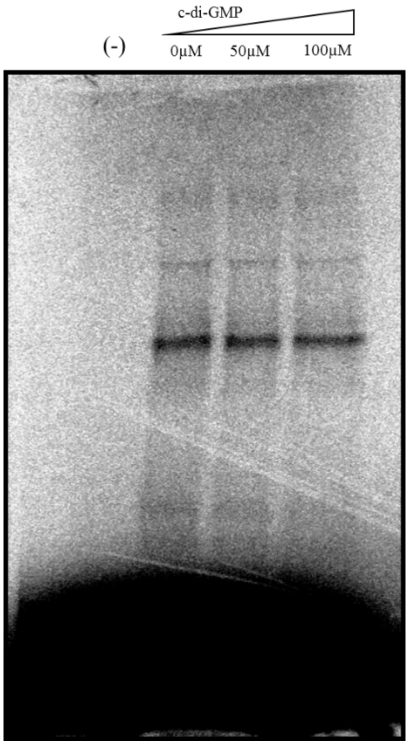

Fig. S2.

I

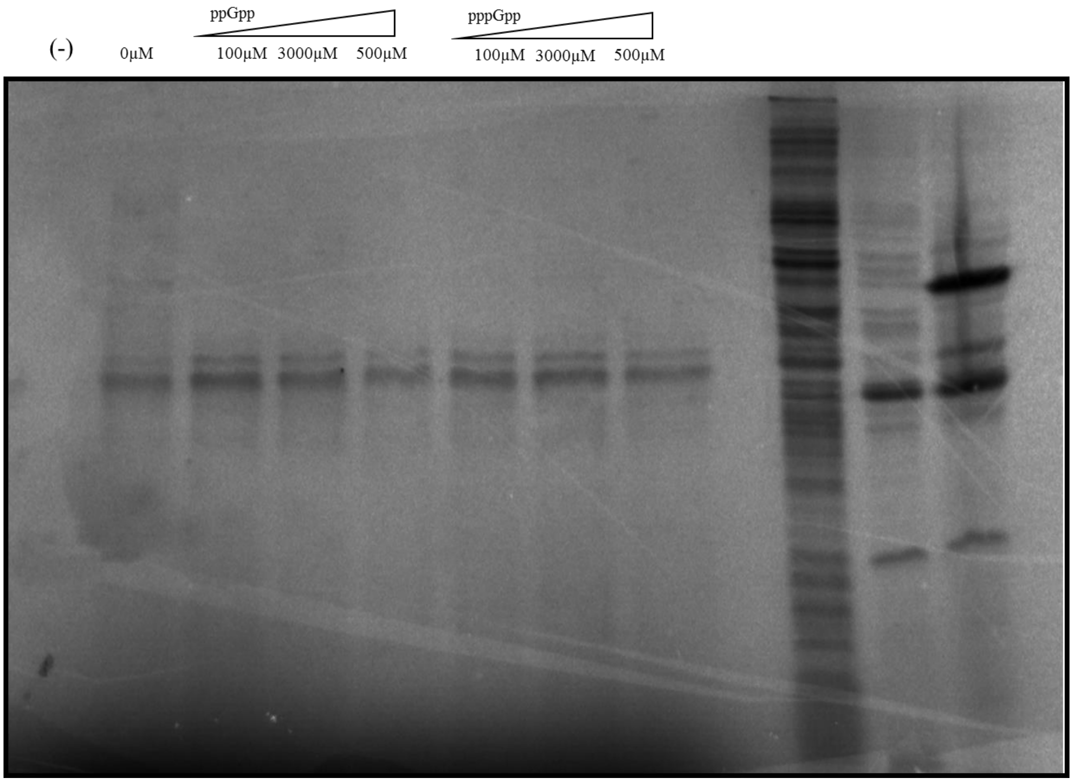

Fig. S2: Analysis of the *ycgG2* gene and its product.

(a) DNA sequence of *ycgG2* split into triplets. The rare start TTG codon is shown in green, the first internal ATG codon – in blue and the stop TGA codon – in red.

(b) Protein alignment of YcgG and Ycg2 showing great similarity between the amino acid residues in the catalytic EAL domain.

(c) Protein alignment between YcgG2 and the protein sequence obtained if the protein is translated from the first internal ATG codon (designated as YcgG2_ATG) showing a 100% match and preservation of the EAL amino acid residues.

(d) Protein alignment between YcgG and YcgG2_ATG showing preservation of the EAL amino acid residues.

(e) Organization of the EAL domain of YcgG2. (Color legend: yellow – residues involved in the EAL domain organization; green – Mg^{2+} - binding residues; blue – c-di-GMP – binding residues; red – glutatamate as a general base catalyst; pink – residues stabilizing the domain).

(f) No effect of c-di-GMP concentrations on YcgG2 translation in *in-vitro* transcription/translation of *ycgG2* under the regulation of T7 promoter (the YcgG2 product shown with an arrow; one representative replica shown of three independent experiments).

(g) No effect of ppGpp and pppGpp alarmones on YcgG2 translation in *in-vitro* transcription/translation of *ycgG2* under the regulation of T7 promoter (the YcgG2 product shown with an arrow; one representative replica shown of three independent experiments).

(h) The full gel of the radioactively labelled YcgG and YcgG2 present in **Fig. 2b** after exposure of 24 hours. The YcgG band was weak and increase of the contrast was applied on the whole picture and then it was used as a main figure.

(i) The full gel of the radioactively labelled YcgG and YcgG2 present in **Fig. 2b** after exposure of 5 days.

(j) The full gel of the radioactively labelled YcgG2 and YcgG2-ATG present in **Fig. 2c** after exposure of 24 hours.

(k) The full gel of the radioactively labelled YcgG2 produced in different concentrations of c-di-GMP present in **Fig. S2f** after exposure of 5 days.

(l) The full gel of the radioactively labelled YcgG2 produced in different concentrations of ppGpp and pppGpp present in **Fig. S2g** after exposure of 5 days.

a

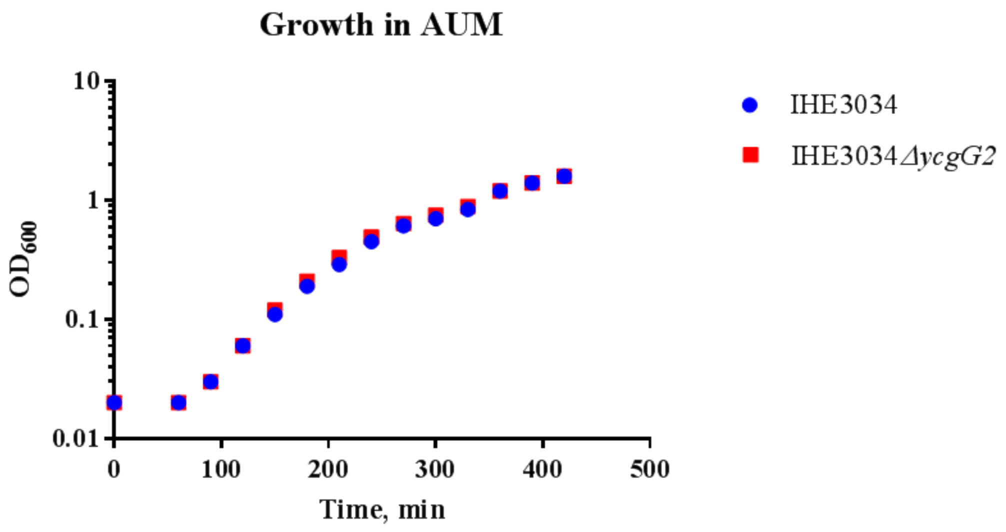
Growth in AUM
10
1
0.1
0.01
OD600
0
100
200
300
400
500
Time, min
IHE3034
IHE3034ΔycgG2

b

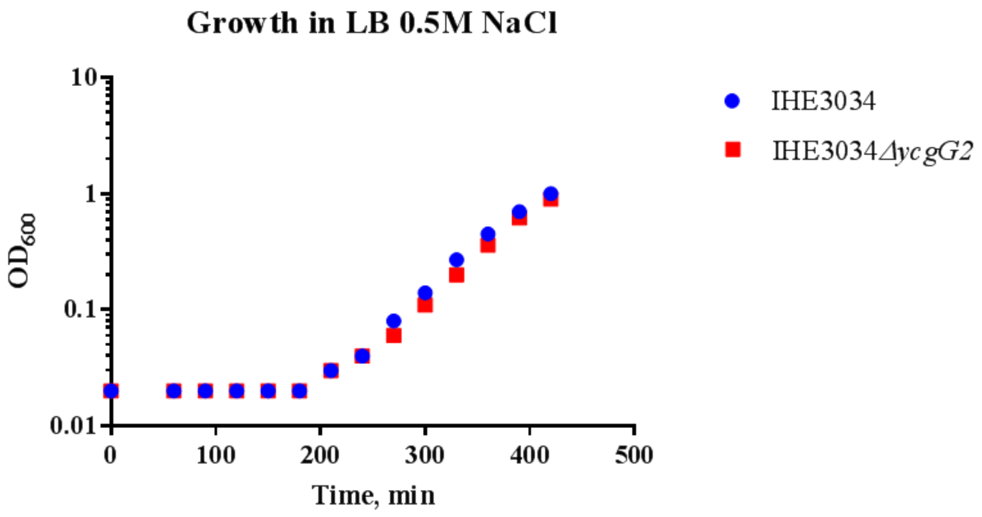
Growth in LB 0.5M NaCl
10
1
0.1
0.01
OD600
0
100
200
300
400
500
Time, min
IHE3034
IHE3034ΔycgG2

Fig. S3

c

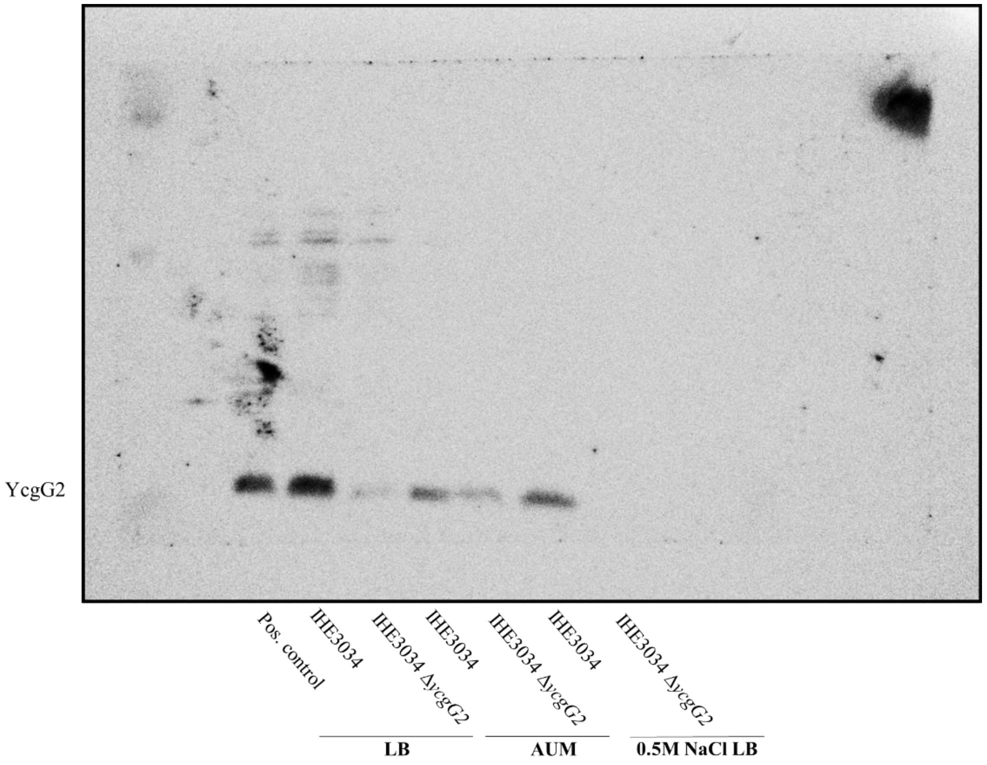
YcgG2
Pos. control
IHE3034
IHE3034 Δ*ycgG2*
IHE3034
IHE3034 Δ*ycgG2*
IHE3034
IHE3034 Δ*ycgG2*
LB
AUM
0.5M NaCl LB

d

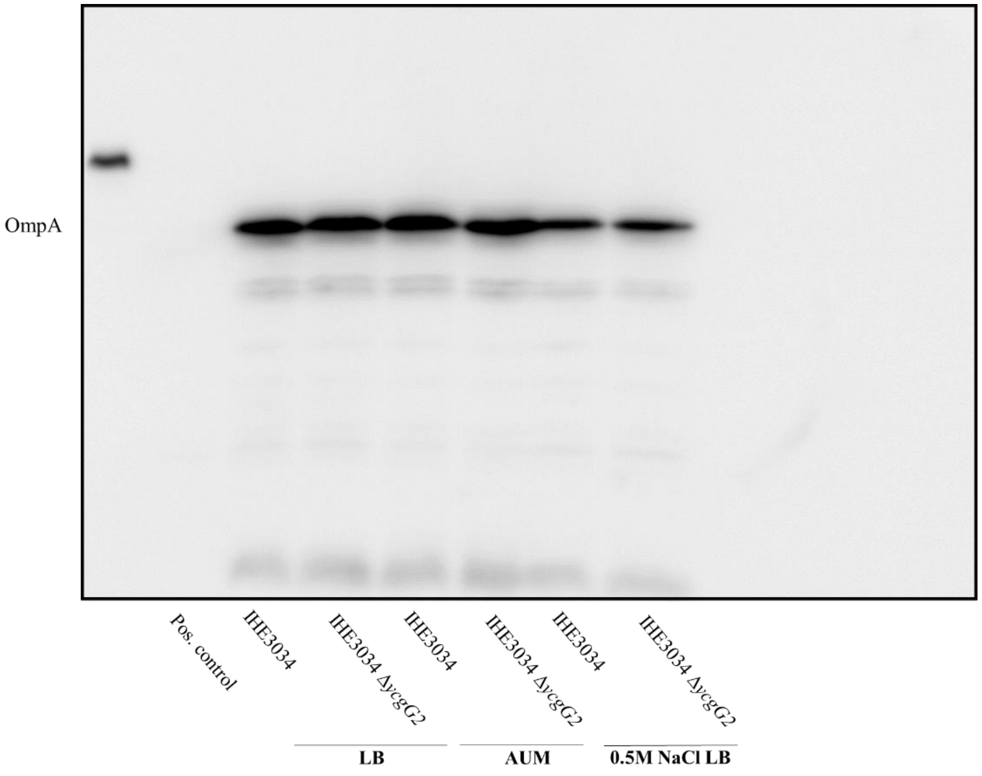
OmpA
Pos. control
IHE3034
IHE3034 Δ*ycgG2*
IHE3034
IHE3034 Δ*ycgG2*
IHE3034
IHE3034 Δ*ycgG2*
LB
AUM
0.5M NaCl LB

Fig. S3

e

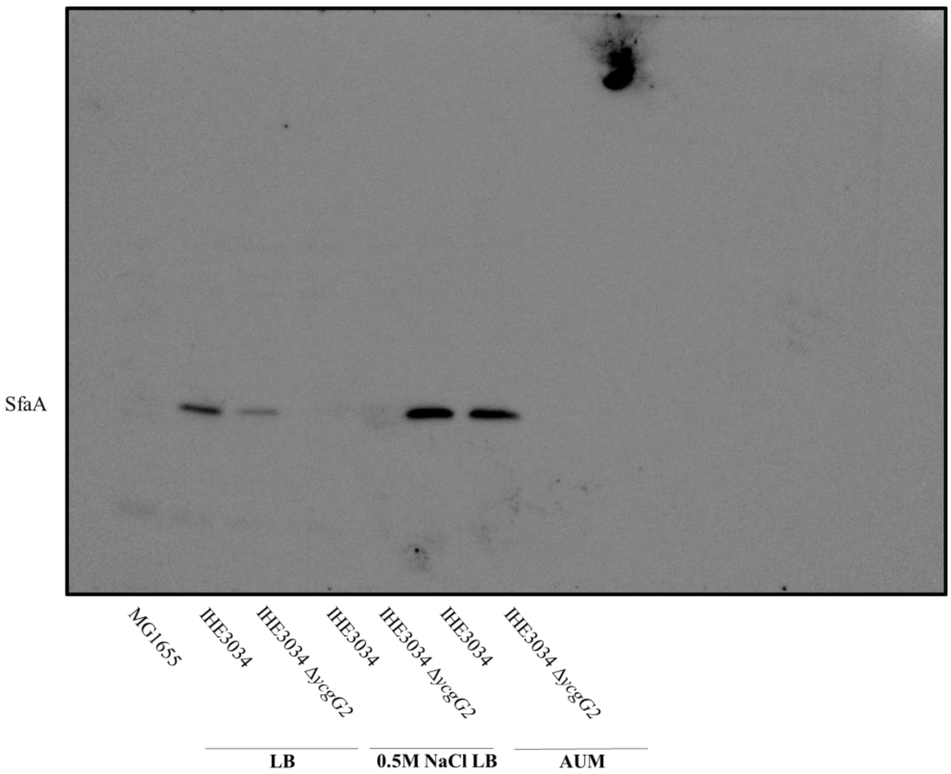
SfaA
MG1655
IHE3034
IHE3034 ΔycgG2
IHE3034
IHE3034 ΔycgG2
IHE3034
IHE3034 ΔycgG2
LB
0.5M NaCl LB
AUM

f

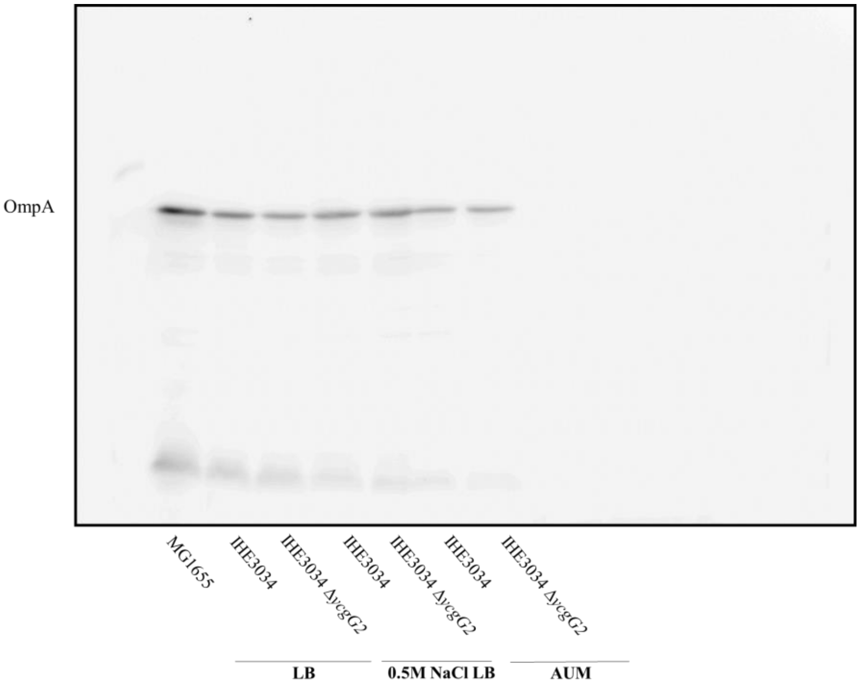
OmpA
MG1655
IHE3034
IHE3034 ΔycgG2
IHE3034
IHE3034 ΔycgG2
IHE3034
IHE3034 ΔycgG2
LB
0.5M NaCl LB
AUM

Fig. S3.

g

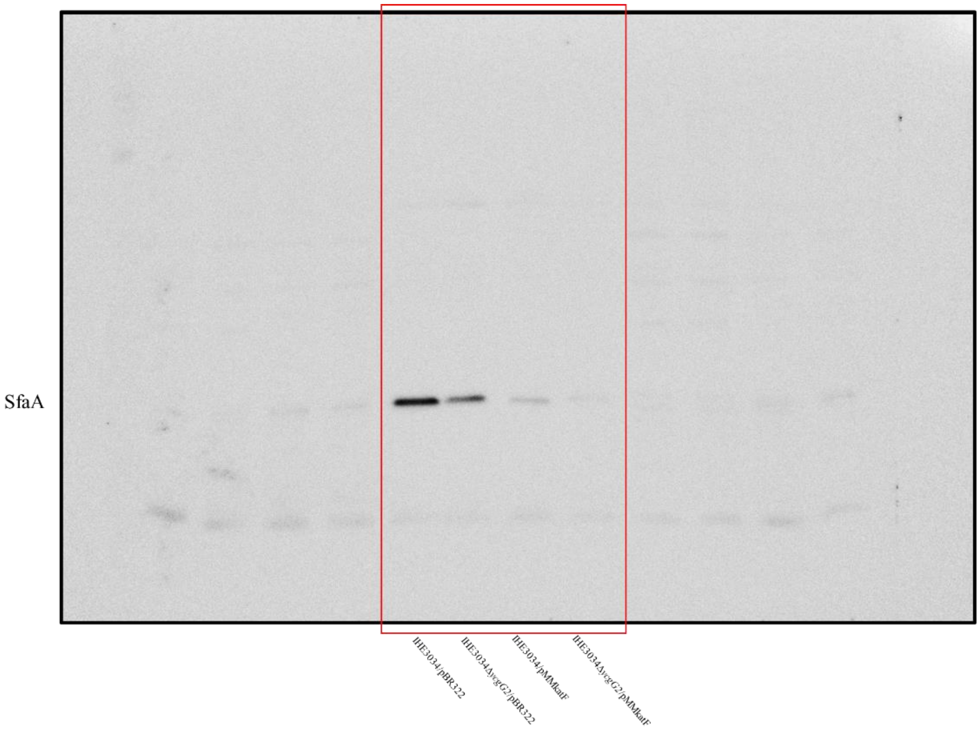
SfaA
IHE3034/pBR322
IHE3034ΔycgG2/pBR322
IHE3034/pMMkatF
IHE3034ΔycgG2/pMMkatF

h

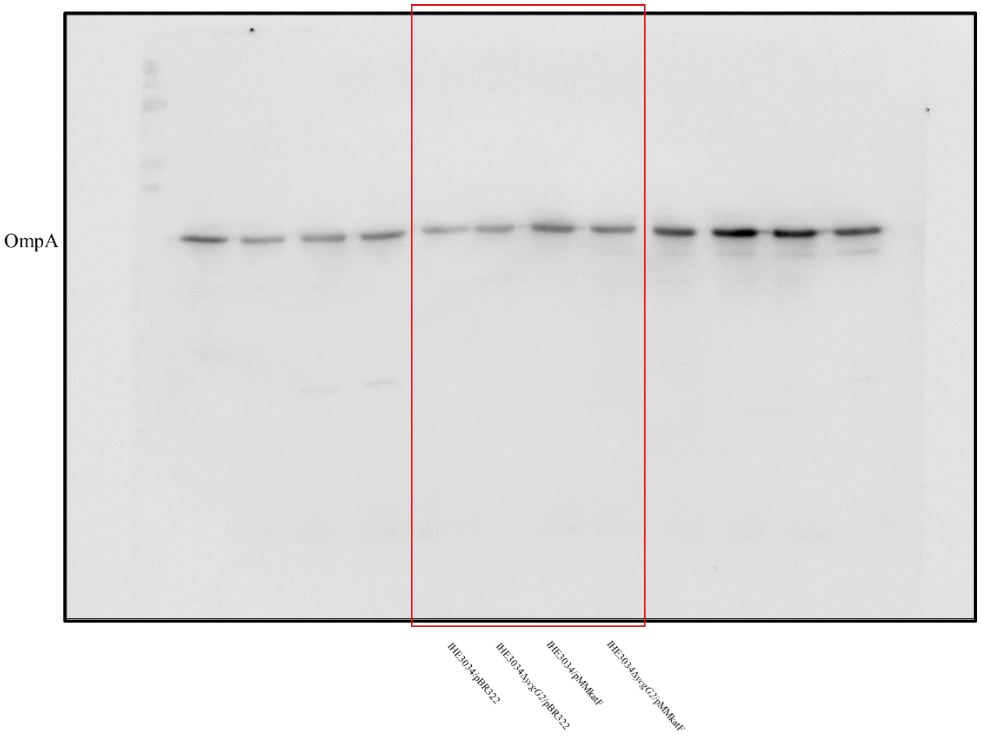
OmpA
IHE3034/pBR322
IHE3034ΔycgG2/pBR322
IHE3034/pMMkatF
IHE3034ΔycgG2/pMMkatF

Fig. S3.

i

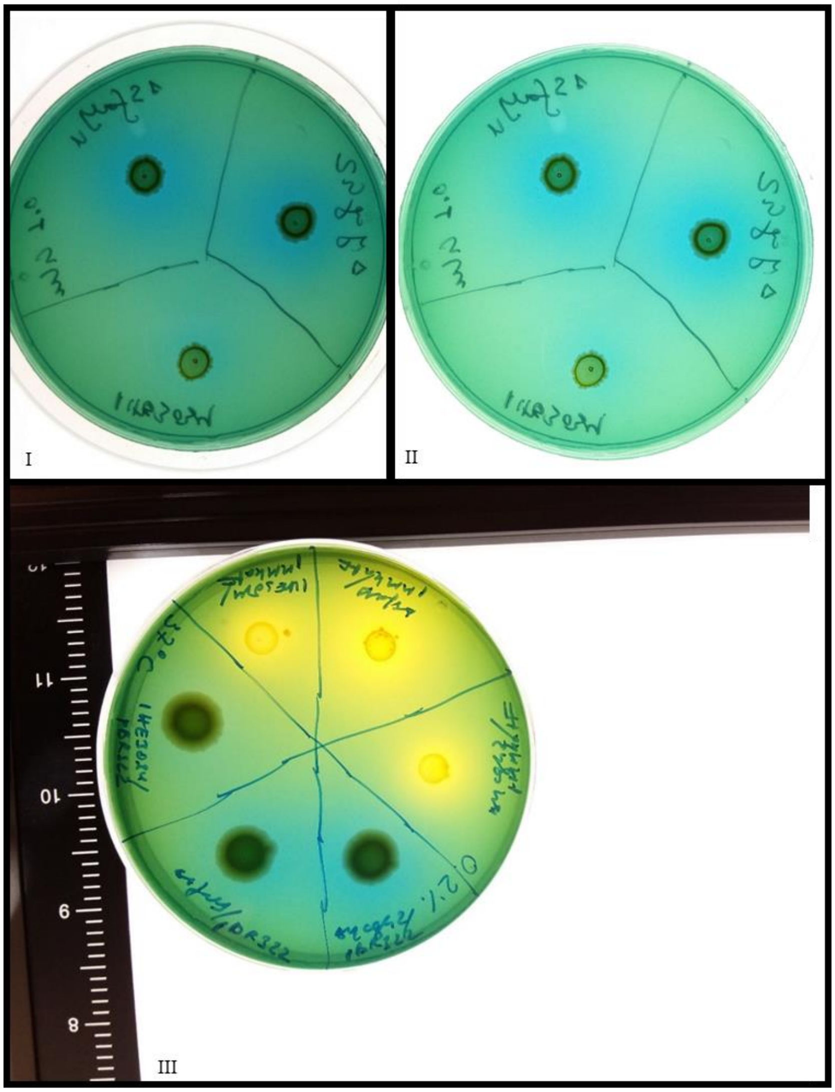

Fig. S3: Properties of the IHE3034 strain and its ycgG2deletion mutant.

No growth difference observed when IHE3034 bacteria and their corresponding Δ*ycgG2* mutant cells were incubated in Artificial Urine Medium (AUM) (a) and in LB supplemented with 0.5M NaCl (b), one representative replica of three independent experiments is shown.

(c) The full membrane of the immunoblot against YcgG2 present in **Fig.3a**.

(d) The full membrane of the immunoblot against OmpA present in **Fig.3a**.

(e) The full membrane of the immunoblot against SfaA present in **Fig. 3b**.

(f) The full membrane of the immunoblot against OmpA present in **Fig.3b.**

(g) The full membrane of the immunoblot against SfaA present in **Fig.3c.**

(h) The full membrane of the immunoblot against OmpA present in **Fig.3c**.

(i) Pictures of Simmon’s plates with cropped labelling present in **Fig.3e** (see Box I) and in **Fig. 3f**.

a

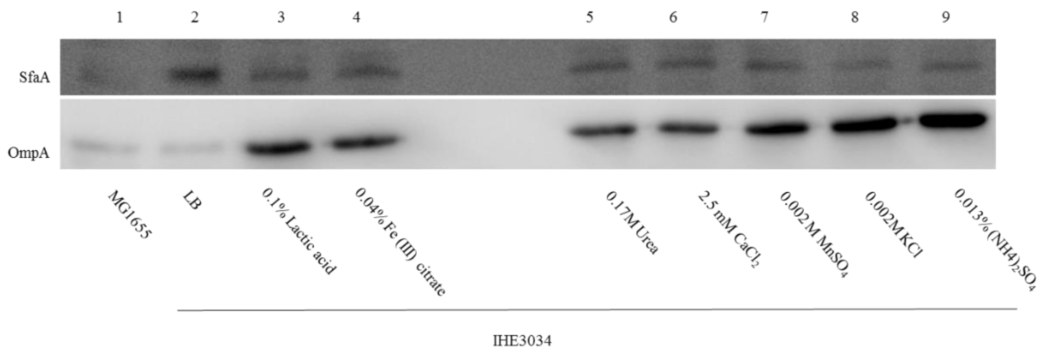
1
2
3
4
5
6
7
8
9
SfaA
OmpA
MG1655
LB
0.1% Lactic acid
0.04%Fe (III) citrate
0.17M Urea
2.5 mM $CaCl_2$
0.002 M $MnSO_4$
0.002M KCl
0.013%$(NH4)_2SO_4$
IHE3034

Fig. S4.

b

SfaA

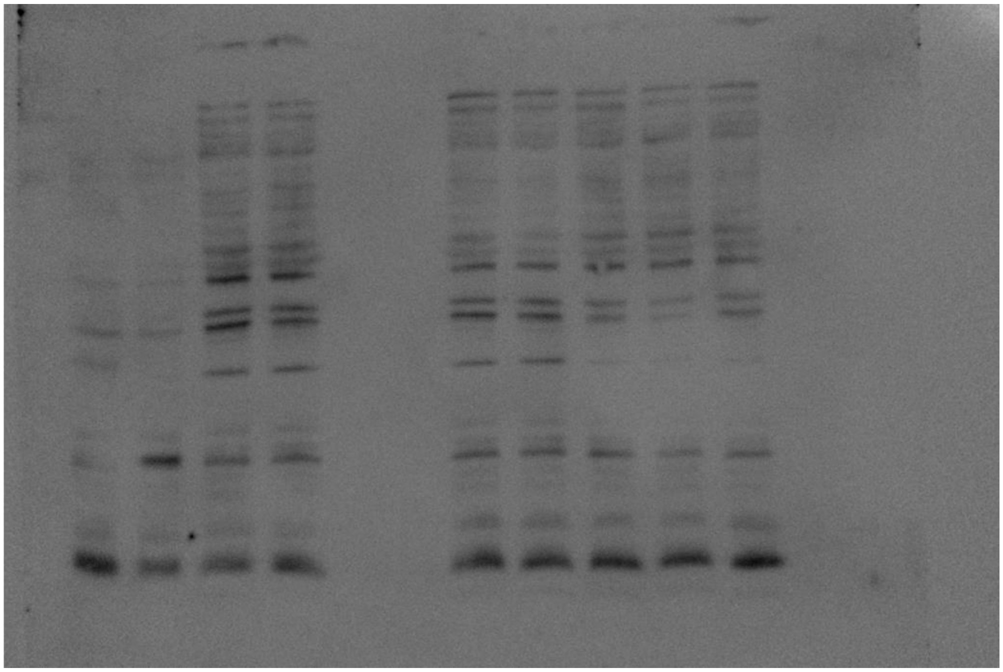

c

OmpA

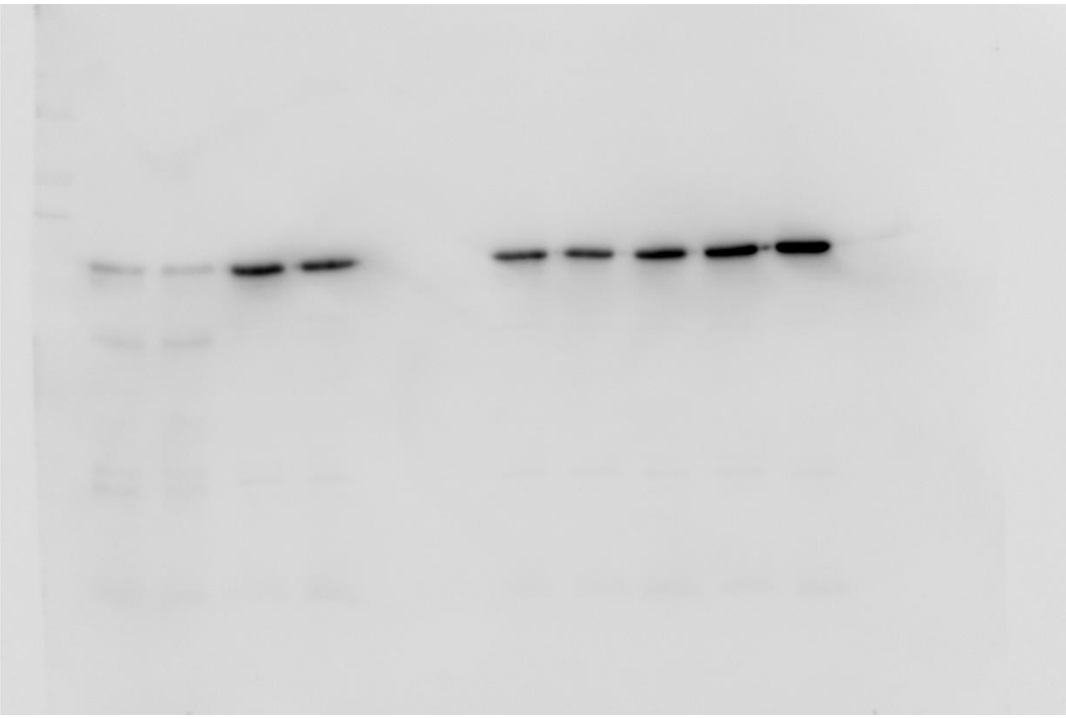

Fig. S4.

Fig. S4: The impact of the different components of the AUM on SfaA production of IHE3034 bacteria.

(a) SfaA production by IHE3034 bacteria incubated in LB (lane 2, positive control), 0.1% lactic acid (lane 3), 0.04% iron (III) citrate (lane 4), 0.17M urea (lane 5), 2.5 mM $CaCl_2$ (lane 6), 0.002 M $MnSO_4$ (lane 7), 0.002 M KCl (lane 8) and 0.013% ammonium sulfate (lane 9). MG1655 protein extract used as a negative control, OmpA levels served as a loading control.

(b) The full membrane of the immunoblot against SfaA present in **Fig. S4a**.

(c) The full membrane of the immunoblot against OmpA present in **Fig. S4a.**

a

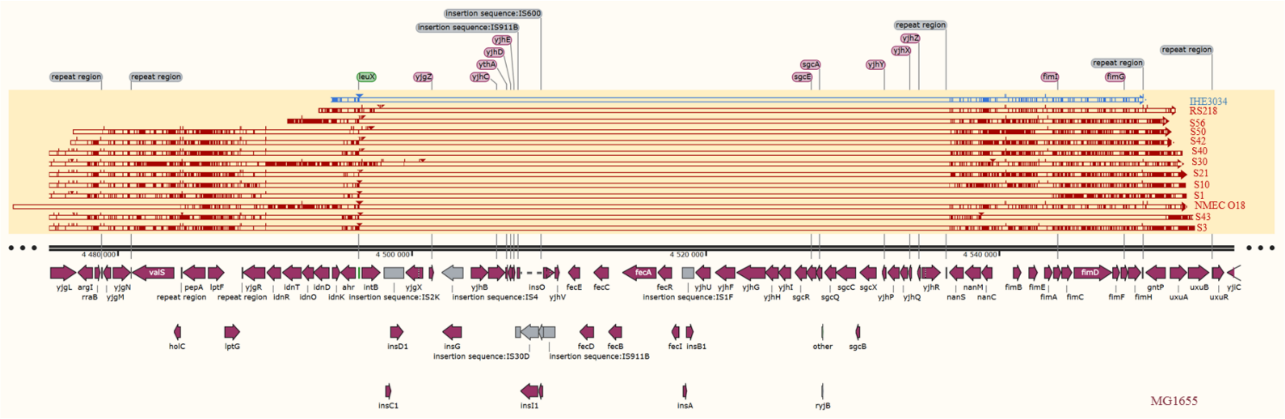
IHE3034
RS218
S56
S50
S42
S40
S30
S21
S10
S1
NMEC O18
S43
S3
MG1655

b

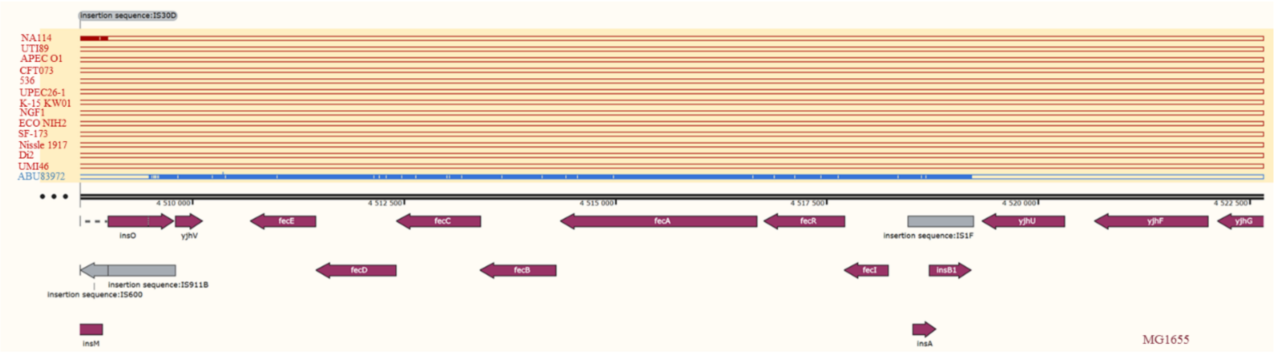
NA114
UTI89
APEC O1
CFT073
536
UPEC26-1
K-15 KW01
NGF1
ECO NIH2
SF-173
Nissle 1917
Di2
UMI46
ABU83972
MG1655

c

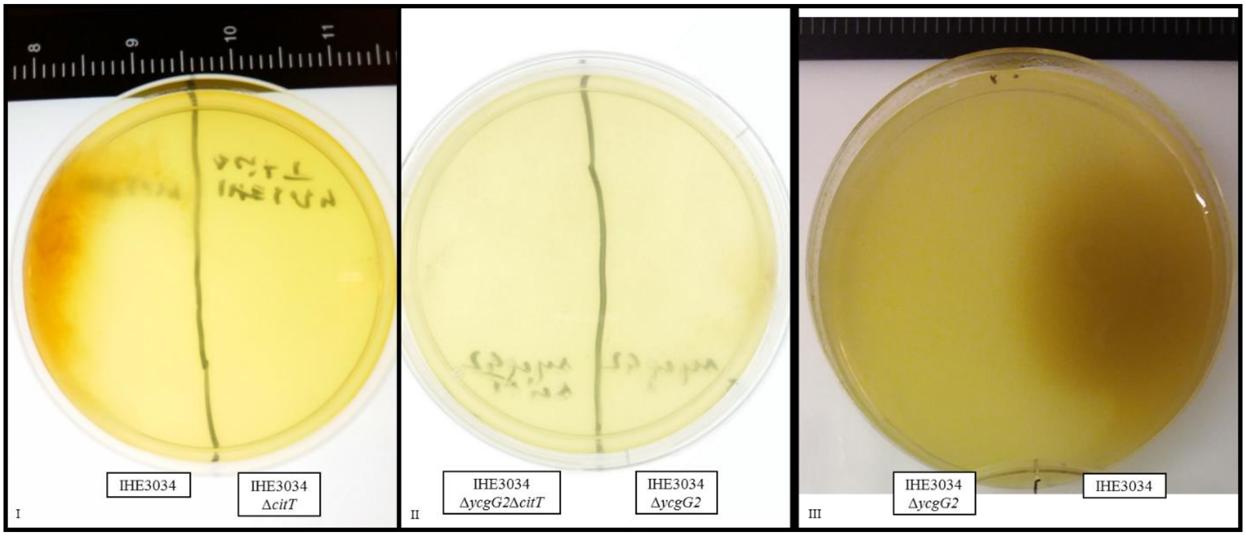
IHE3034
IHE3034 Δ*citT*
I
IHE3034 Δ*ycgG2*Δ*citT*
IHE3034 Δ*ycgG2*
II
IHE3034 Δ*ycgG2*
IHE3034
III

Fig. S5.

Fig. S5: Graphical representation of the local alignment of the deleted ExPEC *fec* genetic region against the one of MG1655 genome.

(a) Local alignment of NMEC sequences (shown in red, the sequence of IHE3034 shown in blue) against the genome of MG1655 (detailed below).

(b) Local alignment of the uropathogenic and avian pathogenic *E. coli* sequences (shown in red) against the genome (shown below) of MG1655. The alignment of the sequence of ABU83972 showed presence of the *fec* gene cluster (shown in blue).

(c) The full pictures of the plates from ferric citrate *in-vitro* assay depicted in **Fig. 5d**.

Table S1. Indel mutations in the genome of IHE3034F compared to the one of IHE3034

IHE3034 Locus/Gene	Position	IHE3034	IHE3034F
ECOK1_0515/phage integrase gene	558439	GTGGGACATATT	G
ECOK1_0536/hypothetical gene	574971	T	TAA
ECOK1_0536/hypothetical gene	574973	TGG	T
ECOK1_0536/hypothetical gene	575051	T	TC
ECOK1_0900/*clpA* (pseudogene due to frameshift)	931694	CGTAAAGA	C
Intergenic region	1161181	C	CG
Intergenic region	1161182	C	CCG
ECOK1_2031/*uvrY*	2054745	G	GGT
Intergenic region	2714858	CGAGTCTTGAGTCTT	C
ECOK1_3036/hypothetical gene	3107961	C	CGTTACCA
ECOK1_3620/hypothetical gene	3716051	CA	C
ECOK1_4442/*btuB* (pseudogene due to frameshift)	4567043	A	AC
ECOK1_4650/*dcuA*	4790789	CG	C
ECOK1_4680/*queG* (pseudogene due to frameshift)	4817885	C	CA
ECOK1_4834/*hyi* (pseudogene due to frameshift)	4976703	C	CA

Table S2. SNPs mutations in the genome of IHE3034F compared to the one of IHE3034

IHE3034 Locus/Gene	Position	IHE3034	IHE3034F
ECOK1_0421/*lon*	463548	T	G
Intergenic region	558394	C	A
ECOK1_0515/phage site-specific recombinase gene	558421	A	T
ECOK1_0515/phage site-specific recombinase gene	558424	C	T
ECOK1_0515/phage site-specific recombinase gene	558427	C	T
ECOK1_0515/phage site-specific recombinase gene	558437	C	G
ECOK1_0536/hypothetical gene	574949	T	C
ECOK1_0536/hypothetical gene	574963	C	T
ECOK1_0536/hypothetical gene	574979	T	C
ECOK1_0536/hypothetical gene	574996	T	C
ECOK1_0536/hypothetical gene	575003	G	C
ECOK1_0536/hypothetical gene	575005	A	C
ECOK1_0536/hypothetical gene	575026	T	C
ECOK1_0536/hypothetical gene	575028	T	C
ECOK1_0536/hypothetical gene	575035	T	C
ECOK1_0536/hypothetical gene	575037	G	A
ECOK1_0536/hypothetical gene	575038	G	A
ECOK1_0536/hypothetical gene	575042	T	C
ECOK1_0536/hypothetical gene	575045	G	A
ECOK1_0536/hypothetical gene	575063	C	T
ECOK1_0536/hypothetical gene	575066	G	A

ECOK1_0536/hypothetical gene	575081	G	A
ECOK1_0536/hypothetical gene	575083	C	T
ECOK1_0536/hypothetical gene	575089	A	T
Intergenic region	605737	A	C
ECOK1_0657/*gltJ*	697000	G	A
ECOK1_0989/*mukF*	1022101	T	C
ECOK1_1066/*putA*	1108192	C	A
ECOK1_1107/*iroN*	1151518	T	C
ECOK1_1107/*iroN*	1151560	T	C
ECOK1_1107/*iroN*	1151581	A	T
ECOK1_1107/*iroN*	1151665	G	A
ECOK1_1107/*iroN*	1151767	C	A
ECOK1_1107/*iroN*	1152044	A	G
ECOK1_1107/*iroN*	1152514	T	C
ECOK1_1108/*iroE*	1154149	T	C
ECOK1_1108/*iroE*	1154179	G	A
ECOK1_1108/*iroE*	1154433	A	G
ECOK1_1109/*iroD*	1154671	T	C
ECOK1_1109/*iroD*	1154728	C	T
ECOK1_1109/*iroD*	1155093	T	C
ECOK1_1109/*iroD*	1155094	G	A
ECOK1_1109/*iroD*	1155388	G	A
ECOK1_1109/*iroD*	1155758	G	A
ECOK1_1110/ABC transporter gene	1155926	C	A
ECOK1_1110/ABC transporter gene	1155974	T	C
ECOK1_1110/ABC transporter gene	1156187	A	G
Intergenic region	1160966	A	G
Intergenic region	1160977	G	A
Intergenic region	1161036	C	T
Intergenic region	1161165	C	T
Intergenic region	1161189	T	A
Intergenic region	1161197	C	T
ECOK1_1112/hypothetical gene	1161357	C	A
ECOK1_1112/hypothetical gene	1161562	C	A
ECOK1_1112/hypothetical gene	1161573	G	T
ECOK1_1112/hypothetical gene	1161575	G	A
ECOK1_1112/hypothetical gene	1161576	T	C
ECOK1_1301/*sitD*	1326729	A	G
ECOK1_1301/*sitD*	1326803	C	T
ECOK1_1301/*sitD*	1326817	T	C
ECOK1_1301/*sitD*	1326872	G	A
ECOK1_1302/*sitC*	1328078	A	G
ECOK1_1303/*sitB*	1328118	A	T

ECOK1_1303/*sitB*	1328313	T	C
ECOK1_1303/*sitB*	1328373	G	C
ECOK1_1303/*sitB*	1328510	T	C
ECOK1_1303/*sitB*	1328547	T	C
ECOK1_1303/*sitB*	1328646	A	C
ECOK1_1304/*sitA*	1328984	C	A
ECOK1_1304/*sitA*	1329032	G	A
ECOK1_1304/*sitA*	1329041	T	C
ECOK1_1304/*sitA*	1329280	T	C
ECOK1_1304/*sitA*	1329341	G	A
ECOK1_1304/*sitA*	1329683	C	A
ECOK1_1304/*sitA*	1329704	C	T
ECOK1_1553/auxiliary transport protein gene	1564992	G	C
ECOK1_2161/hypothetical gene	2187637	C	T
ECOK1_2162/tRNA-Asn gene	2187821	A	G
ECOK1_3005/hypothetical gene	3083132	A	G
ECOK1_3009/phage N-6-adenine-methyltransferase gene	3085261	C	T
ECOK1_3119/*rpoS*, pseudogene	3182422	G	C
ECOK1_3120/*rpoS* (5’ end of the gene before the stop codon)	3182582	C	A
ECOK1_3464/hypothetical gene	3559184	A	G
ECOK1_3519/*sstT*	3620440	G	A
ECOK1_3819/*ompR*	3901439	T	A
ECOK1_4122/*uhpA*	4229388	C	G

Table S3. Bacterial strains used in this work

Strain	Description/Relevant characteristics	Reference/Source
Escherichia coli strains		
MC4100	F$^-$, *(argF-lac)UI69, araOl39, rpsL 150, ptsF25, flbB5301, rbsR, deoC, relA*	[1]
RH90	MC4100 *rpoS359::*Tn10	[2]
BW25113	F- **,** *Δ(araD-araB)567*, *ΔlacZ4787*(::rrnB-3), *λ*$^-$, *rph-1*, *Δ(rhaD-rhaB)568*, *hsdR514*	[3,4]
JW0604-1	BW25113Δ*citT750::kan*	[4]
MG1655	*E. coli* K-12 wild type	[5]
IHE3034	Clinical NMEC isolate, O18:K1:H7	[6]
MAM20	Δ*ycgG2::cat* mutant of IHE3034	This work
AES1	Δ*sfaX::aph* mutant of IHE3034	[7]
AES153	Δ*sfaY* mutant of IHE3034	This work
NZ7	AES153/pBR322	This work
NZ11	IHE3034/pBR322	This work
NZ67	IHE3034/pMMkatF	This work
NZ70	AES153/pMMkatF	This work
NZ215	IHE3034Δ*ycgG2/*pBR322	This work
NZ216	IHE3034Δ*ycgG2/*pMMkatF	This work
NZ217	IHE3034Δ*citT::kan*	This work
NZ218	IHE3034Δ*ycgG2*Δ*citT::kan*	This work
536	Clinical UTI pyelonephritis isolate, O6:K15:H31	[8]
536Δ102	536 (*leuX*$^-$)	[9]
536-21	536 (PAI I$^-$, PAI II$^-$, *selC*$^-$, *leuX*$^-$)	[10]

536R3	536 (PAI I$^-$, PAI II$^-$, *selC*$^-$)	[11]
NZ225	536-21Δ*rpoS::Tn10*	This work
NZ226	536R3Δ*rpoS::Tn10*	This work
UTI89	Clinical UTI cyctitis isolate, O18:K1:H7	[12]
NZ219	UTI89Δ*rpoS::Tn10*	This work
RS218	Clinical NMEC isolate, O18:K1:H7	[13]
DH5α	*ednA1 hsdR17 supE44 thi-1 recA1 gyrA relA1Δ(lacZ4A-argF)*	[14]
IHE1041	Clinical UTI pyelonephritis isolate;<br>O1:K1:H7	Prof. Timo Korhonen,<br>University of Helsinki, Finland
IHE1049	Clinical UTI pyelonephritis isolate;<br>O1:K1:H7	Prof. Timo Korhonen,<br>University of Helsinki, Finland
IHE1402	Clinical UTI pyelonephritis isolate;<br>O6:K2:H1	Prof. Timo Korhonen,<br>University of Helsinki, Finland
IHE1190	Clinical UTI pyelonephritis isolate;<br>O18:K5:H7	Prof. Timo Korhonen,<br>University of Helsinki, Finland
IHE1268	Clinical UTI pyelonephritis isolate;<br>O18:K5:H7	Prof. Timo Korhonen,<br>University of Helsinki, Finland
Vibrio cholerae strains		
C6706	El Tor, Inaba, Str^R	[15]
C6706*luxO*^{c}	C6706, LuxO (L104Q) constitutively active	[16]
NZ44	C6706*luxO*^{c}/pBAD18	This work
NZ92	C6706*luxO*^{c}/pNZK16	This work
NZ93	C6706*luxO*^{c}/pNZK17	This work

Table S4. Plasmids used in this work

Plasmid	Description/Relevant characteristics	Reference/Source
pBAD18	Cb^R, ori ColE1, inducible P_{BAD} promoter	[17]
pBR322	Cb^R, Tc^R, *rep* ori (pMB1), cloning vector	[18]
pMMkatF	Cb^R, 4.2-kb subclone of *rpoS*$_{EC}$ in pAT153	[19]
pNZK16	Cb^R, pBAD18, *ycgG* from MG1655	This work
pNZK17	Cb^R, pBAD18, *ycgG2* from IHE3034	This work
pSIM6	Cb^R, ori SC101, temperature sensitive, carries the α-red recombinase genes, temperature inducible	[20]
pKD3	Cm^R, ori R6Kγ, template for generation of cat fragment flanked with FRT sites.	[3]
pKO3	Cm^R, ori M13, *sacB*, *repA*^{ts}, suicide vector	[21]

Table S5. Primers used in this work

**Primer:**	**Sequence (5'->3'):**
*ycgG2*delFw	TTTATTCTGTTAATCGCCTGCGCCGCTGCCTTCCTGCTTGTGTGTAGGCTGGAGCTGCTTC
*ycgG2*delRv	TGTCTATAATTACACCGCACCATGGCCTTGCTGATCAATTTTCACATATGAATATCCTCCTTAG
1312Fw	AGAGCTCATTCTGCCTACATTGTGCTAAAAAATTAA
*ycgG2*dwdelRv	CCGGATCCTGCCAGCAACCCATTCAGCCGCATACTGGCAGTGAGAAAC
EAL6up	GCGGATCCGGCGGTAATGATATTAC
EALdelendup	CTCTATATTGAGACTCTGCAGCAGGGATAATCCTTTTTCACAGAC
SfaII-3	CGGTCGACGGATCAGCATCACTAGG
EALdelstartdo	CTGCTGCAGAGTCTCAATATAGAGTGACATTACTCCTCCGG
Fw*sfaH*dw	GTGACTTTTAGCTACAACTAGAATGCAG
dd*sfaX*Rv	ATGCGGCCGCTGATAATCAATATCATTTAGCAAAAGAAAAAGCAA
*ycgG*K-12Fw	TGAGCTCCAAGCAAATGCGCAATACAC
*ycgG*K-12Rv	GGGAAGCTTTCACTCAACCACAACCTTCAC
*ycgG*K1FW	TGAGCTCTGGGATTTTGATATTTATTCTGTTAATCGCCTGC
*ycgG*K1RV	GGGAAGCTTTCACTCAACCACAATCTTCACCTTCG
pBAD18FW	ATGCCATAGCATTTTTATCC
pBAD18RV	GATTTAATCTGTATCAGG
T7PmRBS*ycgG*K-12Fw	GAAATTAATACGACTCACTATAGGGAGACCACAACGGTTTCCCTCTAGAAATAATTTTGTTTAACTTTAAGAAGGATATACCAATGCGCAATACACTCATACCCATC
ComplRV*ycgG*	AAGCTTTCACTCAACCACAACCTTCACCTTCGGTTTAGAAAGGATA
T7PmRBS*ycgG*2K1Fw	GAAATTAATACGACTCACTATAGGGAGACCACAACGGTTTCCCTCTAGAAATAATTTTGTTTAACTTTAAGAAGGATATACCATTGATATTTATTCTGTTAATCGCCTGC
up*citT*Fw	AAGCACTTGATAAATTTGGAAATATTAATTTTCGGAGA
dw*citT*Rv	GGGTGTGGAACTCATACATACACTGAA
3120FW	ATGAGTCAGAATACGCTGAAAGTTCA

*rpoS*Rv	TTATTCGCGGAACAGCGCTTCGATATTCA

Supplementary Reference:

1 Gowrishankar, J. Identification of osmoresponsive genes in *Escherichia coli*: evidence for participation of potassium and proline transport systems in osmoregulation. *J Bacteriol* **164**, 434-445 (1985).

2 Marschall, C. *et al.* Molecular analysis of the regulation of *csiD*, a carbon starvation-inducible gene in *Escherichia coli* that is exclusively dependent on sigma s and requires activation by cAMP-CRP. *J Mol Biol* **276**, 339-353, doi:10.1006/jmbi.1997.1533 (1998).

3 Datsenko, K. A. & Wanner, B. L. One-step inactivation of chromosomal genes in *Escherichia coli* K-12 using PCR products. *Proc Natl Acad Sci U S A* **97**, 6640-6645, doi:10.1073/pnas.120163297 (2000).

4 Baba, T. *et al.* Construction of *Escherichia coli* K-12 in-frame, single-gene knockout mutants: the Keio collection. *Mol Syst Biol* **2**, 2006 0008, doi:10.1038/msb4100050 (2006).

5 Guyer, M. S., Reed, R. R., Steitz, J. A. & Low, K. B. Identification of a sex-factor-affinity site in *E. coli* as gamma delta. *Cold Spring Harb Symp Quant Biol* **45 Pt 1**, 135-140 (1981).

6 Korhonen, T. K. *et al.* Serotypes, hemolysin production, and receptor recognition of *Escherichia coli* strains associated with neonatal sepsis and meningitis. *Infect Immun* **48**, 486-491 (1985).

7 Sjöström, A. E. *et al.* The SfaXII protein from newborn meningitis *E. coli* is involved in regulation of motility and type 1 fimbriae expression. *Microb Pathog* **46**, 243-252, doi:10.1016/j.micpath.2009.01.007 (2009).

8 Hacker, J. *et al.* Cloning and characterization of genes involved in production of mannose-resistant, neuraminidase-susceptible (X) fimbriae from a uropathogenic O6:K15:H31 *Escherichia coli* strain. *Infect Immun* **47**, 434-440 (1985).

9 Ritter, A. *et al.* The Pai-associated *leuX* specific tRNA5(Leu) affects type 1 fimbriation in pathogenic *Escherichia coli* by control of FimB recombinase expression. *Mol Microbiol* **25**, 871-882 (1997).

10 Blum, G. *et al.* Excision of large DNA regions termed pathogenicity islands from tRNA-specific loci in the chromosome of an *Escherichia coli* wild-type pathogen. *Infect Immun* **62**, 606-614 (1994).

11 Dobrindt, U., Cohen, P. S., Utley, M., Muhldorfer, I. & Hacker, J. The *leuX*-encoded tRNA5(Leu) but not the pathogenicity islands I and II influence the survival of the uropathogenic *Escherichia coli strain* 536 in CD-1 mouse bladder mucus in the stationary phase. *FEMS Microbiol Lett* **162**, 135-141 (1998).

12 Chen, S. L. *et al.* Identification of genes subject to positive selection in uropathogenic strains of *Escherichia coli*: a comparative genomics approach. *Proc Natl Acad Sci U S A* **103**, 5977-5982, doi:10.1073/pnas.0600938103 (2006).

13 Silver, R. P., Aaronson, W., Sutton, A. & Schneerson, R. Comparative analysis of plasmids and some metabolic characteristics of *Escherichia coli* K1 from diseased and healthy individuals. *Infect Immun* **29**, 200-206 (1980).

14 Hanahan, D. Studies on transformation of *Escherichia coli* with plasmids. *J Mol Biol* **166**, 557-580 (1983).

15 Thelin, K. H. & Taylor, R. K. Toxin-coregulated pilus, but not mannose-sensitive hemagglutinin, is required for colonization by *Vibrio cholerae* O1 El Tor biotype and O139 strains. *Infect Immun* **64**, 2853-2856 (1996).

16 Vance, R. E., Zhu, J. & Mekalanos, J. J. A constitutively active variant of the quorum-sensing regulator LuxO affects protease production and biofilm formation in *Vibrio cholerae*. *Infect Immun* **71**, 2571-2576 (2003).

17 Guzman, L. M., Belin, D., Carson, M. J. & Beckwith, J. Tight regulation, modulation, and high-level expression by vectors containing the arabinose PBAD promoter. *J Bacteriol* **177**, 4121-4130 (1995).

18 Bolivar, F. *et al.* Construction and characterization of new cloning vehicles. II. A multipurpose cloning system. *Gene* **2**, 95-113 (1977).

19 Mulvey, M. R., Sorby, P. A., Triggs-Raine, B. L. & Loewen, P. C. Cloning and physical characterization of *katE* and *katF* required for catalase HPII expression in *Escherichia coli*. *Gene* **73**, 337-345 (1988).

20 Datta, S., Costantino, N. & Court, D. L. A set of recombineering plasmids for gram-negative bacteria. *Gene* **379**, 109-115, doi:10.1016/j.gene.2006.04.018 (2006).

21 Link, A. J., Phillips, D. & Church, G. M. Methods for generating precise deletions and insertions in the genome of wild-type *Escherichia coli*: application to open reading frame characterization. *J Bacteriol* **179**, 6228-6237 (1997).
